# A Multi-Scale Computational Model of Levodopa-Induced Toxicity in Parkinson's Disease

**DOI:** 10.3389/fnins.2022.797127

**Published:** 2022-04-19

**Authors:** Vignayanandam Ravindernath-Jayashree Muddapu, Karthik Vijayakumar, Keerthiga Ramakrishnan, V. Srinivasa Chakravarthy

**Affiliations:** ^1^Department of Biotechnology, Bhupat and Jyothi Mehta School of Biosciences, Indian Institute of Technology Madras, Chennai, India; ^2^Department of Biotechnology, Rajalakshmi Engineering College, Chennai, India

**Keywords:** Parkinson's disease, levodopa, dopamine, substance P, striatum, substantia nigra pars compacta

## Abstract

Parkinson's disease (PD) is caused by the progressive loss of dopaminergic cells in substantia nigra pars compacta (SNc). The root cause of this cell loss in PD is still not decisively elucidated. A recent line of thinking has traced the cause of PD neurodegeneration to metabolic deficiency. Levodopa (L-DOPA), a precursor of dopamine, used as a symptom-relieving treatment for PD, leads to positive and negative outcomes. Several researchers inferred that L-DOPA might be harmful to SNc cells due to oxidative stress. The role of L-DOPA in the course of the PD pathogenesis is still debatable. We hypothesize that energy deficiency can lead to L-DOPA-induced toxicity in two ways: by promoting dopamine-induced oxidative stress and by exacerbating excitotoxicity in SNc. We present a systems-level computational model of SNc-striatum, which will help us understand the mechanism behind neurodegeneration postulated above and provide insights into developing disease-modifying therapeutics. It was observed that SNc terminals are more vulnerable to energy deficiency than SNc somas. During L-DOPA therapy, it was observed that higher L-DOPA dosage results in increased loss of terminals in SNc. It was also observed that co-administration of L-DOPA and glutathione (antioxidant) evades L-DOPA-induced toxicity in SNc neurons. Our proposed model of the SNc-striatum system is the first of its kind, where SNc neurons were modeled at a biophysical level, and striatal neurons were modeled at a spiking level. We show that our proposed model was able to capture L-DOPA-induced toxicity in SNc, caused by energy deficiency.

## Introduction

Levodopa (L-DOPA), a precursor of dopamine (DA), is used as a symptom-relieving treatment for Parkinson's disease (PD) (Poewe et al., 2010). The usage of L-DOPA for PD is still debated due to its side effects with long-term treatment (Thanvi and Lo, 2004; Fahn, 2005; Lipski et al., 2011). Several researchers suggested that L-DOPA might be harmful to SNc cells by a mechanism that probably involves oxidative stress (Pardo et al., 1995; Carvey et al., 1997; Takashima et al., 1999). However, several others proposed that L-DOPA might not accentuate neurodegeneration of SNc neurons (Jenner and Brin, 1998; Fahn et al., 2004; Fahn, 2005; Billings et al., 2019) and sometimes acts as a neuroprotective agent (Fahn, 2005; Schapira, 2008; Shimozawa et al., 2019) or promotes recovery of dopaminergic markers in the striatum (Murer et al., 1998, 1999). After several studies, it is still not clear why L-DOPA is not toxic in the case of nonparkinsonian human subjects and healthy animals and toxic in PD models of rodents (Fahn, 1997; Ziv et al., 1997; Agid, 1998; Müller et al., 2004; Weiner, 2006; Lipski et al., 2011; Olanow and Obeso, 2011; Paoletti et al., 2019). The beneficial or toxic effects of L-DOPA need to be investigated with more thorough experiments performed at preclinical and clinical levels.

Almost all neurodegenerative diseases have a characteristic loss of a certain special type of cells that are vulnerable to death due to metabolic deficiency (Fu et al., 2018; Muddapu et al., 2020). PD is characterized by loss of dopaminergic neurons in the substantia nigra pars compacta (SNc), which results in cardinal symptoms, such as tremor, rigidity, bradykinesia, and postural instability (Goldman and Postuma, 2014). The root cause of SNc cell loss in PD is still not decisively elucidated. Recent studies have proposed that PD is described to be resulting from the metabolic deficiency in SNc (Connolly et al., 2014; Pacelli et al., 2015; Muddapu et al., 2019; Muddapu and Chakravarthy, 2020). The vulnerable cells of SNc are projection neurons with large axonal arbors of complex morphologies, requiring a tremendous amount of energy to maintain information processing activities (Bolam and Pissadaki, 2012; Pissadaki and Bolam, 2013; Giguère et al., 2019; Muddapu et al., 2020). Due to substantial energy requirements, SNc neurons exhibit higher basal metabolic rates and higher oxygen consumption rates, which result in oxidative stress (Pacelli et al., 2015). With the help of computational models, Muddapu and co-workers have recently suggested that the excitotoxic loss of SNc cells might be due to energy deficiency occurring at different levels of neural hierarchy—systems, cellular and subcellular (Muddapu et al., 2019; Muddapu and Chakravarthy, 2020, 2021).

In this article, we investigated using computational modeling, the hypothesis that L-DOPA-induced toxicity can occur in two ways: by promoting DA-induced oxidative stress (autoxidation-relevant; Pardo et al., 1995; Walkinshaw and Waters, 1995; Carvey et al., 1997; Melamed et al., 1998; Takashima et al., 1999; Borah and Mohanakumar, 2010) or by exacerbating excitotoxicity in SNc (autoxidation-irrelevant; Pardo et al., 1993; Cheng et al., 1996; Blomeley and Bracci, 2008; Blomeley et al., 2009; Thornton and Vink, 2015), or by both the mechanisms, which might be precipitated by energy deficiency. To investigate our hypothesis, we propose a multiscale computational model of L-DOPA-induced toxicity in SNc, which will help us understand the mechanism behind neurodegeneration due to L-DOPA and give insights into developing disease-modifying therapeutics.

## Materials and Methods

**Figure 1:**
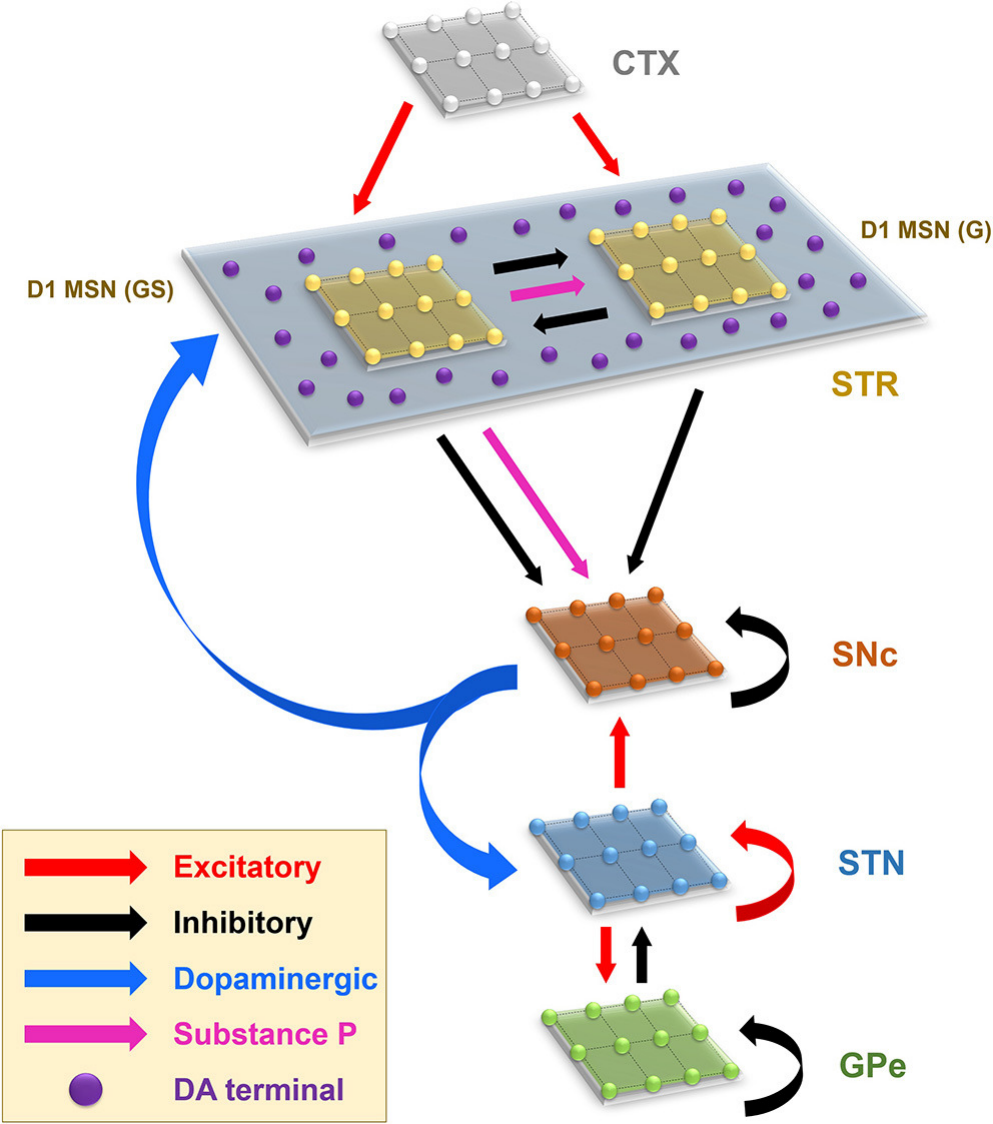
Model architecture of the levodopa-induced toxicity. CTX, cortex; STR, striatum; D1 MSN (GS), D1-type medium spiny neuron (GABAergic and Substance P); D1 MSN (G), D1-type medium spiny neuron (GABAergic); SNc, substantia nigra pars compacta; STN, subthalamic nucleus; GPe, globus pallidus externa; DA, dopamine.

The proposed model of L-DOPA-induced toxicity (LIT) consists of the cortico-basal ganglia system. We modeled a part of the basal ganglia system, comprising the following nuclei: SNc, striatum (STR), subthalamic nucleus (STN), and globus pallidus externa (GPe). Within the SNc, we separately considered SNc soma (cell body) and SNc terminal (bouton) that make contact with striatal neurons. The medium spiny neurons (MSNs) in the striatum are classified into two types based on the DA receptor present, namely, D1 and D2-types. In the proposed LIT model, we considered only D1-type MSNs because they only project GABAergic inputs to SNc neurons (Gerfen, 1985). Within the striatum, we modeled D1-type receptor-expressing MSNs of two subtypes: (1) D1-MSNs that release GABA only [D1-MSN (G)] [GABAergic (G) only] and (2) D1-MSNs that co-release GABA and substance P [D1-MSN (GS)] [GABAergic (G), and substance P (S)]. The pyramidal neurons in the cortex are classified into three types: regular spiking (RS), intrinsic (inactivating) bursting, and non-inactivating bursting neurons. The regular spiking neurons are further subdivided into fast-adapting and slow-adapting types of neurons (Degenetais, 2002). The time-averaged firing rate of all neuronal types varies widely, ranging from < 1 *Hz* up to several tens of hertz (Griffith and Horn, 1966; Koch and Fuster, 1989). The spontaneous firing rates of all pyramidal cortical neuronal types are as follows: fast-adapting RS (0.62 ±0.75 *Hz*), slow-adapting RS (0.90 ±1.23 *Hz*), intrinsic bursting (3.1 ±2.6 *Hz*), and non-inactivating bursting (2.8 ±3.2 *Hz*) (Degenetais, 2002). In the cortex (CTX), the fast-adapting RS pyramidal neurons are modeled. Neurons in each nucleus are arranged as a two-dimensional lattice (Figure 1). The simulations were performed by numerical integration using MATLAB (RRID:SCR_001622) with a time step of 0.1*ms*.

Glucose and oxygen inputs to SNc cells were reduced to implement energy deficiency conditions in the proposed model. As the number of SNc neurons under energy deficiency increases, the dopaminergic tone to the striatum decreases due to SNc terminal loss. The dopamine deficiency leads to lesser excitation of MSN neurons by pyramidal neurons in the cortex; as a result, SNc neurons get disinhibited. Disinhibition from MSN leads to overactivity of SNc neurons, resulting in SNc neurons degeneration due to excitotoxicity.

To examine L-DOPA's role in the degeneration of SNc neurons in PD, we administered L-DOPA after a certain percentage of SNc neuronal loss due to energy deficiency and investigated how L-DOPA changes the course of SNc cell loss.

### Izhikevich (Spiking) Neuron Model (STN, GPe, MSN, and CTX)

The Izhikevich neuron models can exhibit biologically realistic firing patterns at a relatively low computational expense (Izhikevich, 2003). The proposed model of LIT consists of MSN [D1-MSN (G) and D1-MSN (GS)], STN, GPe, and CTX. These nuclei are modeled as Izhikevich spiking neuron models arranged in the form of two-dimensional lattices (Figure 1). Based on the anatomical data of the rat basal ganglia (Supplementary Material 1), the neuronal population sizes in the model were selected (Oorschot, 1996; Arbuthnott and Wickens, 2007). The Izhikevich parameters for MSN were adapted from Humphries et al. (2009); for STN and GPe, they were adapted from Michmizos and Nikita (2011) and Mandali et al. (2015), respectively, and those of CTX were adapted from Izhikevich (2003), all given in Supplementary Material 2. The external bias current (*I*^*x*^) was adjusted to match the firing rate of nuclei with published data (Tripathy et al., 2015).

The Izhikevich neuron model of STN, GPe, CTX, and MSN neurons consists of two variables: membrane potential (*v*^*x*^) and membrane recovery variable (*u*^*x*^):


(1) $$\begin{array}{l} C^{x}*\frac{d\left(v_{ij}^{x}\right)}{dt}=\varphi_{1}-u_{ij}^{x}+I_{ij}^{x}+I_{ij}^{syn} \end{array}$$

(2) $$\begin{array}{l} \frac{d\left(u_{ij}^{x}\right)}{dt}=a\left(b*\varphi_{2}-u_{ij}^{x}\right) \end{array}$$

(3) $$\begin{array}{l} if\;x=STN\;or\;GPe\;or\;CTX,\;then\\ \;\varphi_{1}=0.04{\left(v_{ij}^{x}\right)}^{2}+5v_{ij}^{x}+140\\ \;\varphi_{2}=v_{ij}^{x} \end{array}$$

(4) $$\begin{array}{l} if\;x=MSN,\;then\\ \;\varphi_{1}=k^{x}\left(v_{ij}^{x}-v_{r}^{x}\right)\left(v_{ij}^{x}-v_{t}^{x}\right)\\ \;\varphi_{2}=\left(v_{ij}^{x}-v_{r}^{x}\right) \end{array}$$

Resetting:


(5) $$\begin{array}{l} if\;v_{ij}^{x}\geq v_{peak}^{x},\;then\;\left[\begin{array}{c} v_{ij}^{x}\leftarrow c\\ u_{ij}^{x}\leftarrow u_{ij}^{x}+d \end{array}\right] \end{array}$$

where, $v_{ij}^{x}$, $u_{ij}^{x}$, $I_{ij}^{x}$, and $I_{ij}^{syn}$ are the membrane potential, the membrane recovery variable, the external bias current, and the total synaptic current received to neuron *x* at the location (*i, j*), respectively;${\;v}_{t}^{x}$ and $v_{r}^{x}$ are the threshold and resting potentials, respectively; *k*^*x*^ is the membrane constant, *C*^*x*^ is the membrane capacitance, {*a, b, c, d*} are Izhikevich parameters;${\;v}_{peak}^{x}$ is the maximum membrane voltage set to neuron with *x* being GPe or CTX or STN or MSN neuron.

### Biophysical Neuron Model (SNc Soma)

The biophysical neuronal model of SNc in the proposed LIT model was adapted from Muddapu and Chakravarthy (2021). The detailed biophysical model of SNc neuron consists of soma and terminal, which includes cellular and molecular processes, such as ion channels (including active ion pumps and ion exchangers), a calcium buffering mechanism (calcium-binding proteins, calcium sequestration organelles, such as endoplasmic reticulum and mitochondria), an energy metabolism (glycolysis and oxidative phosphorylation), DA turnover processes (synthesis, storage, release, reuptake, and metabolism), molecular pathways involved in PD pathology (reactive oxygen species (ROS) formation and alpha-synuclein aggregation), and apoptotic pathways. The dynamics of SNc membrane potential (*v*^*SNc*^) is given as,


(6) $$\begin{array}{l} \frac{d\left(v_{ij}^{SNc}\right)}{dt}=\frac{F*v_{cyt}}{C^{SNc}{*A}_{pmu}}*\left[J_{m,Na}+2*J_{m,Ca}+J_{m,K}+J_{inp}\right] \end{array}$$

where *F* is the Faraday's constant, *C*^*SNc*^ is the SNc membrane capacitance, *v*_*cyt*_ is the cytosolic volume, $A_{pmu}$ is the cytosolic area, *J*_*m,Na*_ is the sodium membrane ion flux, *J*_*m,Ca*_ is the calcium membrane ion flux, *J*_*m,K*_ is the potassium membrane ion flux, *J*_*inp*_ is the overall input current flux. The detailed information about the SNc neuron model is provided in Supplementary Material 3.

### Biochemical Dopamine Terminal Model (SNc Terminal)

The biochemical DA terminal of SNc in the proposed LIT model was adapted from Muddapu and Chakravarthy (2021). The biochemical model of DA terminal consists of DA turnover processes, energy metabolism, and molecular pathways involved in PD pathology. The terminal is divided into two compartments, namely, intracellular (cytoplasmic and vesicular) and extracellular compartments. The DA dynamics in the extracellular compartment ([*DA*_*e*_]) was modeled as,


(7) $$\begin{array}{l} \frac{d\left(\left[DA_{e}\right]\right)}{dt}=J_{rel}-J_{DAT}-J_{eda}^{o} \end{array}$$

where, *J*_*rel*_ represents the flux of calcium-dependent DA release from the DA terminal, *J*_*DAT*_ represents the unidirectional flux of DA translocated from the extracellular compartment (ECS) into the intracellular compartment (cytosol) *via* DA plasma membrane transporter (DAT), and $J_{eda}^{o}$ represents the outward flux of DA degradation, which clears DA from ECS.

The DA dynamics in the intracellular compartment ([*DA*_*i*_]) was modeled as


(8) $$\begin{array}{l} \frac{d\left(\left[DA_{i}\right]\right)}{dt}=\frac{d\left(\left[DA_{c}\right]\right)}{dt}+\frac{d\left(\left[DA_{v}\right]\right)}{dt} \end{array}$$

where [*DA*_*c*_] and [*DA*_*v*_] refer to the DA concentrations in the cytosolic and vesicular compartments, respectively.

The DA dynamics in the cytosolic compartment ([*DA*_*c*_]) is given by,


(9) $$\begin{array}{l} \frac{d\left(\left[DA_{c}\right]\right)}{dt}=J_{DAT}-J_{VMAT}-J_{cda}^{o}+J_{ldopa} \end{array}$$

where, *J*_*DAT*_ represents the unidirectional flux of DA translocated from ECS into the cytosol through DAT, *J*_*VMAT*_ represents the flux of DA into vesicle through vesicular monoamine transporters (VMAT), $J_{ida}^{o}$ represents the outward flux of DA degradation, which clears DA from the cytosol, and *J*_*ldopa*_ represents the flux of synthesized cytosol DA from levodopa, which is induced by calcium.

The DA dynamics in the vesicular compartment ([*DA*_*v*_]) is given by,


(10) $$\begin{array}{l} \frac{d\left(\left[DA_{v}\right]\right)}{dt}=J_{VMAT}-J_{rel} \end{array}$$

where, *J*_*rel*_ represents the flux of calcium-dependent DA release from the DA terminal and *J*_*VMAT*_ represents the flux of DA stored into a vesicle.

Based on the membrane activity, the DA turnover and other molecular processes were modulated in the terminal. The modulation of neuronal activity on the terminal was carried on by calcium dynamics, where calcium modulates DA synthesis and release. The calcium-induced synthesis of DA is given as,


(11) $$\begin{array}{l} J_{ldopa}=f\left(\left[Ca_{i}\right]\right) \end{array}$$

The calcium-induced release of DA is given as,


(12) $$\begin{array}{l} J_{rel}=f\left(\left[Ca_{i}\right]\right) \end{array}$$

where [*Ca*_*i*_] is the intracellular calcium concentration in the DA terminal. Detailed information about the SNc terminal model was provided in Supplementary Material 4.

### Synaptic Connections

The synaptic connectivity among different neuronal populations was modeled as a standard single exponential model of postsynaptic currents (Humphries et al., 2009) as follows:


(13) $$\begin{array}{l} \tau_{Recep}*\frac{d\left(h_{ij}^{x\to y}\right)}{dt}=-h_{ij}^{x\to y}+S_{ij}^{x}\;(t) \end{array}$$

(14) $$\begin{array}{l} I_{ij}^{x\to y}\;(t)=W_{x\to y}*h_{ij}^{x\to y}\;(t)*\left(v_{ij}^{y}\;(t)-E_{Recep}\right) \end{array}$$

The N-Methyl-D-aspartic Acid (NMDA) current was regulated by voltage-dependent magnesium channels, which were modeled as,


(15) $$\begin{array}{l} B_{ij}\left(v_{ij}\right)=\frac{1}{1+\left(\frac{\left[Mg^{2+}\right]}{3.57}*e^{-{0.062\;*v}_{ij}^{y}\;(t)}\right)} \end{array}$$

where, $h_{ij}^{x\to y}$ is the gating variable for the synaptic current from *x* to *y*, τ_*Recep*_ is the decay constant for the synaptic receptor, $S_{ij}^{x}$ is the spiking activity of neuron *x* at time *t*, *W*_*x*→*y*_ is the synaptic weight from neuron *x* to *y*, $v_{ij}^{y}$ is the membrane potential of the neuron *y* for the neuron at the location (*i, j*), *E*_*Recep*_ is the receptor-associated synaptic potential (*Recep* = NMDA/AMPA/GABA), and [*Mg*^2+^] is the magnesium ion concentration. The time constants of gamma-amino butyric acid (GABA), alpha-amino-3-hydroxy-5-methyl-4-isoxazole propionic acid (AMPA), and NMDA in GPe, CTX, MSN, SNc, and STN were chosen from Götz et al. (1997) as given in Supplementary Material 2.

To accommodate extensive axonal arborization of SNc neurons (Bolam and Pissadaki, 2012), we considered one-to-many projections from SNc soma to SNc terminals (Supplementary Material 5). The connectivity patterns among different neuronal populations were given in Supplementary Material 5.

### Total Synaptic Current Received by Each Neuron Type

#### SNc

The total synaptic current received by an *SNc* neuron at the lattice position (*i, j*) is the summation of the glutamatergic input from the *STN* neurons, considering both *NMDA* and *AMPA* receptor activation, comprising the GABAergic inputs from the *D*1 − *MSN* (*GS*) and *D*1 − *MSN* (*G*) neurons and the lateral GABAergic current from other *SNc* neurons.


(16) $$\begin{array}{l} I_{ij}^{SNcsyn}=F_{STN\to SNc}*\left(I_{ij}^{NMDA\to SNc}+\;I_{ij}^{AMPA\to SNc}\right)\\ \;+\left(F_{D1-MSN\;\left(G\right)\to SNc}*I_{ij}^{D1-MSN\left(G\right)\to SNc}\right)\\ \;+\left(F_{D1-MSN\;\left(GS\right)\to SNc}*I_{ij}^{D1-MSN\left(GS\right)\to SNc}\right)+I_{ij}^{GABAlat} \end{array}$$

where $I_{ij}^{NMDA\to SNc}$ and $I_{ij}^{AMPA\to SNc}$ are the glutamatergic currents corresponding to *NMDA* and *AMPA* receptors activation, respectively; $I_{ij}^{D1-MSN\left(G\right)\to SNc}$ and $I_{ij}^{D1-MSN\left(GS\right)\to SNc}$ are the GABAergic inputs from the *D*1 − *MSN* (*G*) and *D*1 − *MSN* (*GS*) neurons, respectively; $I_{ij}^{GABAlat}$ is the lateral GABAergic current from other *SNc* neurons; *F*_*STN*→*SNc*_ is the scaling factor in the glutamatergic current from the *STN* neuron; *F*_*D*1 − *MSN* (*G*) → *SNc*_ is the scaling factor in the GABAergic current from *D*1 − *MSN* (*G*) neuron; *F*_*D*1 − *MSN* (*GS*) → *SNc*_ is the scaling factor in the GABAergic current from the *D*1 − *MSN* (*GS*) neuron.

#### GPe

The total synaptic current received by a *GPe* neuron at the lattice position (*i, j*) is the summation of the glutamatergic input from the *STN* neurons, considering both *NMDA* and *AMPA* receptors activation and the lateral GABAergic current from other *GPe* neurons.


(17) $$\begin{array}{l} I_{ij}^{GPesyn}=I_{ij}^{NMDA\to GPe}+I_{ij}^{AMPA\to GPe}+I_{ij}^{GABAlat} \end{array}$$

where $I_{ij}^{NMDA\to GPe}$ and ${\;I}_{ij}^{AMPA\to GPe}$ are the glutamatergic currents from the *STN* neuron, considering both *NMDA* and *AMPA* receptors activation, respectively; $I_{ij}^{GABAlat}$ is the lateral GABAergic current from other *GPe* neurons.

#### STN

The total synaptic current received by an *STN* neuron at the lattice position (*i, j*) is the summation of the GABAergic input from the *GPe* neurons and the lateral glutamatergic input from other *STN* neurons, considering both *NMDA* and *AMPA* receptors activation.


(18) $$\begin{array}{l} I_{ij}^{STNsyn}=I_{ij}^{GABA\to STN}+I_{ij}^{NMDAlat}+I_{ij}^{AMPAlat} \end{array}$$

where $I_{ij}^{GABA\to STN}$ is the GABAergic current from the *GPe* neuron; $I_{ij}^{NMDAlat}$ and $I_{ij}^{AMPAlat}$ are the lateral glutamatergic currents from other *STN* neurons, considering both *NMDA* and *AMPA* receptors activation, respectively.

#### D1-MSN (GS)

The total synaptic current received by a *D*1 − *MSN* (*GS*) neuron at the lattice position (*i, j*) is the summation of the GABAergic input from the *D*1 − *MSN* (*G*) neurons and the glutamatergic input from *CTX* neurons, considering both *NMDA* and *AMPA* receptors activation.


(19) $$\begin{array}{l} I_{ij}^{D1-MSN\left(GS\right)syn}=I_{ij}^{GABA\to D1-MSN\left(GS\right)}+I_{ij}^{NMDA\to D1-MSN\left(GS\right)}\\ \;+I_{ij}^{AMPA\to D1-MSN\left(GS\right)} \end{array}$$

where $I_{ij}^{GABA\to D1-MSN\left(GS\right)}$ is the GABAergic current from the *D*1 − *MSN*(*G*) neuron, $I_{ij}^{NMDA\to D1-MSN\left(GS\right)}$, and $I_{ij}^{AMPA\to D1-MSN\left(GS\right)}$ are the glutamatergic currents from *CTX* neurons, considering both *NMDA* and *AMPA* receptors activation, respectively.

#### D1-MSN (G)

The total synaptic current received by a *D*1 − *MSN* (*G*) neuron at the lattice position (*i, j*) is the summation of the GABAergic input from the *D*1 − *MSN* (*GS*) neurons and the glutamatergic input from *CTX* neurons, considering both *NMDA* and *AMPA* receptor activation.


(20) $$\begin{array}{l} I_{ij}^{D1-MSN\left(G\right)syn}=I_{ij}^{GABA\to D1-MSN\left(G\right)}+I_{ij}^{NMDA\to D1-MSN\left(G\right)}\\ \;+I_{ij}^{AMPA\to D1-MSN\left(G\right)} \end{array}$$

where $I_{ij}^{GABA\to D1-MSN\left(G\right)}$ is the GABAergic current from the *D*1 − *MSN*(*GS*) neuron, and $I_{ij}^{NMDA\to D1-MSN\left(G\right)}$, and $I_{ij}^{AMPA\to D1-MSN\left(G\right)}$ are the glutamatergic currents from *CTX* neurons, considering both *NMDA* and *AMPA* receptors activation, respectively.

### Lateral Connections

The lateral connections in SNc, STN, and GPe were modeled as Gaussian neighborhoods (Muddapu et al., 2019),


(21) $$\begin{array}{l} w_{ij,pq}^{m\to m}=A_{m}*e^{\frac{-d_{ij,pq}^{2}}{R_{m}^{2}}} \end{array}$$

(22) $$\begin{array}{l} d_{ij,pq}^{2}={\left(i-p\right)}^{2}+{\left(j-q\right)}^{2} \end{array}$$

where $w_{ij,pq}^{m\to m}$ is the lateral connection weight of neuron type *m* at the location (*i, j*), *d*_*ij,pq*_ is the distance from the center neuron (*p, q*), *R*_*m*_ is the variance of Gaussian, and *A*_*m*_ is the strength of lateral synapse, *m* = *GPe or STN or SNc*.

The connections within SNc and GPe populations were considered inhibitory and within STN as excitatory (Muddapu et al., 2019) (Figure 1). No lateral connections were considered for both the MSNs and CTX populations. The lateral currents in the STN and GPe were modeled similar to Equations 13–15 and in the case of SNc, which was modeled as,


(23) $$\begin{array}{l} H_{\infty }=\frac{1}{1+e^{\left(\frac{-\left(v_{ij}^{x}-\theta_{g}-\theta_{g}^{H}\right)}{\sigma_{g}^{H}}\right)}} \end{array}$$

(24) $$\begin{array}{l} \frac{d\left(s_{ij}^{x\to y}\right)}{dt}=\alpha *\left(1-s_{ij}^{x\to y}\right)*H_{\infty }-\beta *s_{ij}^{x\to y} \end{array}$$

(25) $$\begin{array}{l} I_{ij}^{x\to y}\;(t)=W_{x\to y}*s_{ij}^{x\to y*}\left(v_{ij}^{y}\;(t)-E_{GABA}\right) \end{array}$$

where $I_{ij}^{x\to y}$ is the synaptic current from neuron *x* to *y*, *W*_*x*→*y*_ is the synaptic conductance from neurons *x* to *y*, $v_{ij}^{x}$ and $v_{ij}^{y}$ are the membrane potential of the neurons *x* and *y*, respectively, for the neuron at the location (*i, j*), *E*_*GABA*_ is the GABAergic receptor potential, and $s_{ij}^{x\to y}$ is the synaptic gating variable for the neuron. The parametric values of α, β, θ_*g*_, $\theta_{g}^{H}$, $\sigma_{g}^{H}$ were adapted from Rubin and Terman (2004) and given in Supplementary Material 6.

### Neuromodulatory Effect on the Neuronal Populations

The effect of neuromodulators, such as DA and substance P (SP), in the proposed LIT model was modeled based on Buxton et al. (2017) and Muddapu et al. (2019), respectively.

#### Dopaminergic Modulation

DA-modulated lateral connection strength in SNc, STN, and GPe populations. As the DA level increases, the lateral connection strength in SNc and GPe increases, whereas, in the case of STN, it decreases (Kreiss et al., 1997). DA-modulation of lateral connection strength was modeled as,


(26) $$\begin{array}{l} A^{STN}=s_{\max}^{STN}*e^{\left(-cd_{stn}\;*DA_{s}\left(t\right)\right)} \end{array}$$

(27) $$\begin{array}{l} A^{GPe}=s_{\min}^{GPe}*e^{\left(cd_{gpe}\;*DA_{s}\left(t\right)\right)} \end{array}$$

(28) $$\begin{array}{l} A^{SNc}=s_{\min}^{SNc}*e^{\left(cd_{snc}\;*DA_{s}\left(t\right)\right)} \end{array}$$

where $s_{\max}^{STN}$, $s_{\min}^{GPe}$, and $s_{\min}^{SNc}$ are lateral connection strengths at the basal spontaneous activity of the population without any external dopaminergic influence in *STN*, *GPe*, and *SNc*, respectively. *cd*_*stn*_, *cd*_*gpe*_, and *cd*_*snc*_ were the factors by which DA affects the lateral connections in *STN*, *GPe*, and *SNc* populations, respectively, and *DA*_*s*_ (*t*) is the instantaneous DA level, the spatial average DA concentration of all the terminals. All parameter values are given in Supplementary Material 6.

The post-synaptic effect of DA in SNc, STN, and GPe was modeled similar to Muddapu et al. (2019),


(29) $$\begin{array}{l} W_{x\to y}=\left(1-cd2*DA_{s}\;(t)\right)*w_{x\to y} \end{array}$$

where *w*_*x* → *y*_ is the synaptic weight (*STN* → *GPe, GPe* → *STN, STN* → *STN, GPe* → *GPe, STN* → *SNc, SNc* → *SNc, MSN* → *SNc*), *cd*2 is the parameter that affects the post-synaptic current, and *DA*_*s*_ (*t*) is the instantaneous DA level.

The effect of DA in the MSN population occurs on both synaptic and intrinsic ion channels (Surmeier et al., 2007). The cortical inputs to MSN were modulated by DA as similar to Humphries et al. (2009),


(30) $$\begin{array}{l} I_{DA}^{x}\;(t)=I_{CTX\to MSN}^{x}\;(t)*\left(1+\left(\frac{\beta_{DA}}{\alpha_{DA}^{y}}\right)*DA_{s}\;(t)\right) \end{array}$$

where $I_{CTX\to MSN}^{x}$ is the synaptic current from *CTX* to *MSN* (where *x* = *NMDA or AMPA*), *DA*_*s*_ (*t*) is the instantaneous DA level, $\alpha_{DA}^{y}$ is the DA effect on the *y* neuron [where *y* = *D*1 − *MSN* (*GS*) *or D*1 − *MSN* (*G*))], and β_*DA*_ was adapted from Humphries et al. (2009).

In addition to modulating cortical afferent connections, DA also has effects on the intrinsic ion channels (Humphries et al., 2009), which was modeled in the Izhikevich neuron model as,


(31) $$\begin{array}{l} v_{r}^{DA}=v_{r}^{MSN*}\left(1+K^{MSN}*\left(\frac{DA_{s}\;(t)}{\alpha_{DA}^{y}}\right)\right) \end{array}$$

(32) $$\begin{array}{l} d_{msn}^{DA}=d_{msn}*\left(1-L^{MSN}*\left(\frac{DA_{s}\;(t)}{\alpha_{DA}^{y}}\right)\right) \end{array}$$

where $v_{r}^{DA}$ and $d_{msn}^{DA}$ are the DA-modulated resting potential and after-spike reset value of *MSN*, respectively, $v_{r}^{MSN}$, and *d*_*msn*_ are the resting potentials and after-spike reset value of *MSN*, respectively, *DA*_*s*_ (*t*) is the instantaneous DA level;${\;\alpha }_{DA}^{y}$ is the DA effect on the *y* neuron [where *y* = *D*1 − *MSN* (*GS*) *or D*1 − *MSN* (*G*))], and *K*^*MSN*^ and *L*^*MSN*^ were adapted from Humphries et al. (2009).

#### Substance P Modulation

SP modulates excitatory afferent connections of SNc (soma) and D1 MSN (G) in the proposed LIT model (Figure 1). It was observed that SP modulates the glutamatergic afferents of MSNs directly (Blomeley and Bracci, 2008) or indirectly (Blomeley et al., 2009) by co-release of SP by GABAergic D1 MSNs (Reiner et al., 2010; Buxton et al., 2017). In the proposed LIT model, we modeled SP-modulation of glutamatergic afferents of the D1 MSN (G) population by the D1 MSN (GS) population similar to Buxton et al. (2017). It was observed that SP and tachykinin NK1 receptor (NK1-R) are highly expressed within the SNc (Mantyh et al., 1984; Sutoo et al., 1999; Ribeiro-da-Silva and Hökfelt, 2000; Lessard and Pickel, 2005; Thornton and Vink, 2015). SP-containing striatal neurons project to dopaminergic neurons where SP potentiates the release of striatal DA (Brimblecombe and Cragg, 2015; Thornton and Vink, 2015). It was reported that a DA-dependent decrease in SP levels was observed in the basal ganglia regions (Sivam, 1991; Thornton et al., 2010; Thornton and Vink, 2015). Therefore, there is a feedback regulation between DA and SP, which helps maintain DA homeostasis (Thornton et al., 2010; Thornton and Vink, 2015). In the proposed LIT model, we assumed that SP modulates STN glutamatergic inputs to SNc such that increased SP levels lead to excitation of SNc, which, in turn, enhances the striatal DA level, modeled similar to Buxton et al. (2017). Also, we incorporated SP-DA feedback regulation (SDFR) in SP-modulation in the proposed LIT model. The SP-modulation of glutamatergic inputs to D1 MSN (G) and SNc along with SDFR was given as,


(33) $$\begin{array}{l} I_{ij}^{x\to y}\;(t)=W_{x\to y}*h_{ij}^{x\to y}\;(t)*NSP*SDFR*\left(v_{ij}^{y}\;(t)-E_{Recep}\right) \end{array}$$

(34) $$\begin{array}{l} NSP=\left(1+{w_{sp}*N}_{ij}^{sp}\left(t-\tau_{d}^{sp}\right)\right) \end{array}$$

(35) $$\begin{array}{l} N_{ij}^{sp}\;(t)=\beta_{sp}*\left[1-e^{{\left(\frac{-A_{ij}^{sp}\;(t)}{\lambda_{sp}}\right)}^{b_{sp}}}\right] \end{array}$$

(36) $$\begin{array}{l} A_{ij}^{sp}\;(t)=\left[e^{\left(\frac{-S_{ij}^{x}\;(t)}{\tau_{f}^{sp}}\right)}-e^{\left(\frac{-S_{ij}^{x}\;(t)}{\tau_{r}^{sp}}\right)}\right] \end{array}$$

(37) $$\begin{array}{l} SDFR=\left(1-DA_{s}\;(t)\right) \end{array}$$

where $h_{ij}^{x\to y}$ is the gating variable for the synaptic current from *x* to *y*, *W*_*x*→*y*_ is the synaptic weight from neurons *x* to *y*, $S_{ij}^{x}$ is the spiking activity of neuron *x* at time *t*, $v_{ij}^{y}$ is the membrane potential of the neuron *y* for the neuron at the location (*i, j*), *E*_*Recep*_ is the receptor-associated synaptic potential (*Recep* = NMDA/AMPA), $\tau_{d}^{sp}$ is the fixed time delay between MSN activity and the onset of neuropeptide effect, β_*sp*_ is the gain factor, $N_{ij}^{sp}$ is the modulatory effect of SP, *w*_*sp*_ is the influence of SP on *w*_*STN*→*SNc*_, $A_{ij}^{sp}$ is the amplitude of SP released, which is induced by spiking activity $\left(S_{ij}^{x}\right)$, *DA*_*s*_ (*t*) is the instantaneous DA level, *b*_*sp*_ and λ_*sp*_ were adapted from Buxton et al. (2017) and given in Supplementary Material 6.

### Neurodegeneration of SNc Neurons

Calcium plays a dual role in living organisms as a survival factor or a ruthless killer (Orrenius et al., 2003). For the survival of neurons, minimal (physiological) levels of glutamate stimulation are required. Under normal conditions, calcium concentration within a cell is tightly regulated by pumps, transporters, calcium-binding proteins, endoplasmic reticulum (ER), and mitochondria (Wojda et al., 2008; Surmeier et al., 2011). Due to prolonged calcium influx driven by excitotoxicity, the calcium homeostasis within the cell is disturbed, which results in cellular imbalance, leading to the activation of apoptotic pathways (Bano and Ankarcrona, 2018). The SNc soma undergoes degeneration when a calcium build-up inside the cell becomes high, resulting in calcium loading inside ER and mitochondria, which leads to ER-stress-induced and mitochondrial-induced apoptosis, respectively (Malhotra and Kaufman, 2011). In the proposed LIT model, we incorporate a mechanism of programmed cell death, whereby an SNc neuron under high stress (high calcium levels) kills itself. The stress in a given SNc neuron was observed by monitoring the intracellular calcium concentrations in the cytoplasm, ER, and mitochondria.

The SNc neuron undergoes ER-stress-induced apoptosis when calcium levels in ER cross a certain threshold (*ER*_*thres*_). Under such conditions, the particular SNc neuron gets eliminated as follows,


(38) $$\begin{array}{l} if\;Ca_{ij}^{ER}\;(t)>ER_{thres},\;then\;v_{ij}^{SNc}\;(t)=0 \end{array}$$

where $Ca_{ij}^{ER}$ is the calcium concentration in the ER, *ER*_*thres*_ is the calcium concentration threshold after which ER-stress-induced apoptosis gets initiated $\left(ER_{thres}=2.15\;x\;10^{-3}\;mM\right)$, and $v_{ij}^{SNc}$ is the membrane voltage of the neuron at the lattice position (*i, j*).

The ER calcium concentration ([*Ca*_*er*_]) dynamics is given by,


(39) $$\begin{array}{l} \frac{d\left(\left[Ca_{er}\right]\right)}{dt}=\frac{\beta_{er}}{\rho_{er}}*\left(J_{serca,er}-J_{ch,er}-J_{leak,er}\right) \end{array}$$

where β_*er*_ is the ratio of free calcium to total calcium concentration in the ER, ρ_*er*_ is the volume ratio between the ER and cytosol, *J*_*serca,er*_ is the calcium buffering flux by ER uptake of calcium through SERCA, *J*_*ch,er*_ is the calcium efflux from ER by CICR mechanism, and *J*_*leak,er*_ is the calcium leak flux from ER. The detailed information about the calcium dynamics in ER was provided in Supplementary Material 8.

The SNc neuron undergoes mitochondria-induced apoptosis when calcium levels in mitochondria cross a certain threshold (*MT*_*thres*_). Then, that particular SNc neuron will be eliminated as follows,


(40) $$\begin{array}{l} if\;Ca_{ij}^{MT}\;(t)>MT_{thres},\;then\;v_{ij}^{SNc}\;(t)=0 \end{array}$$

where $Ca_{ij}^{MT}$ is the calcium concentration in mitochondria, *MT*_*thres*_ is the calcium concentration threshold beyond which mitochondria-induced apoptosis gets initiated (*MT*_*thres*_ = 0.0215 *mM*), and $v_{ij}^{SNc}$ is the membrane voltage of neurons at the lattice position (*i, j*).

The MT calcium concentration ([*Ca*_*mt*_]) dynamics is given by,


(41) $$\begin{array}{l} \frac{d\left(\left[Ca_{mt}\right]\right)}{dt}=\frac{\beta_{mt}}{\rho_{mt}}*\left(J_{mcu,mt}-J_{out,mt}\right) \end{array}$$

where β_*mt*_ is the ratio of free calcium to total calcium concentration in the ER, ρ_*mt*_ is the volume ratio between the MT and cytosol, *J*_*mcu,mt*_ is the calcium buffering flux by MT uptake of calcium through MCUs, and *J*_*out,mt*_ is the calcium efflux from MT through sodium-calcium exchangers, mPTPs, and non-specific leak flux. The detailed information about the calcium dynamics in MT is provided in Supplementary Material 8.

When calcium concentration in ER crosses a certain threshold, there is an efflux of excess calcium from ER out into the cytoplasm, which activates calpain and proapoptotic factors through the cytochrome-c-independent apoptotic pathway. Similarly, when calcium concentration in MT crosses a certain threshold, excess calcium in MT results in the formation of mitochondrial transition pores (MTPs). Proapoptotic cytochrome-c is released from MT through MTPs, which triggers cytochrome-c-dependent apoptosis. In the proposed modeling study, when the apoptotic signal gets activated from either of the pathways in a particular neuron, we formulated an approach wherein that particular neuron was eliminated by making $v_{ij}^{SNc}\;(t)=0$ from the time *t* till the end of the simulation.

### Terminal Degeneration of SNc Neurons

DA is the primary contributor to the oxidative stress in the neuron (Luo and Roth, 2000; Lotharius et al., 2005; Miyazaki and Asanuma, 2008). To evade oxidative stress, SNc neurons tightly regulate the DA turnover processes (Guo et al., 2018). It was inferred that methamphetamine-induced dopaminergic nerve terminal loss (Ricaurte et al., 1982, 1984; Cadet et al., 2003; Ares-Santos et al., 2014) is precipitated by oxidative stress (De Vito and Wagner, 1989) by enhancing cytoplasmic DA levels (Larsen et al., 2002; Mark et al., 2004). In the proposed LIT model, the oxidative stress in the SNc terminals was observed by monitoring intracellular ROS concentration. The SNc terminal is eliminated when ROS levels in the terminal cross a certain threshold (*ROS*_*thres*_) as follows,


(42) $$\begin{array}{l} if\;\left[ROS_{ij}^{T}\right]\;(t)>ROS_{thres},\;then\;Ca_{ij}^{T}\;(t)=0 \end{array}$$

where $ROS_{ij}^{T}$ is the ROS concentration in the SNc terminal, *ROS*_*thres*_ is the ROS concentration threshold above which oxidative stress-induced terminal degeneration gets initiated (*ROS*_*thres*_ = 0.0147 *mM*);${\;Ca}_{ij}^{T}$ is the calcium concentration of the SNc terminal at the lattice position (*i, j*).

The ROS concentration in the SNc terminal was given as,


(43) $$\begin{array}{l} \frac{d\left(\left[ROS_{ij}^{T}\right]\right)}{dt}=J_{leak}+J_{env}+J_{dopa}-J_{cat}-J_{dox} \end{array}$$

where *J*_*leak*_ is the flux of oxidative stress due to mitochondrial leakage, *J*_*env*_ is the flux of external oxidative stress (includes environmental toxins, inflammatory responses, etc.), *J*_*dopa*_ is the flux of oxidative stress due to excess cytoplasmic dopamine, *J*_*cat*_ is the catabolizing flux of ROS by a catalase enzyme, and *J*_*dox*_ is the flux of the GSH-dependent ROS-scavenging pathway. The detailed information about the ROS formation is provided in Supplementary Material 9.

When the ROS level crosses a certain threshold, excess ROS triggers degeneration of the terminal. In the proposed modeling study, when the ROS level crosses the threshold in a particular terminal, we formulate an approach wherein that particular terminal was eliminated by making $Ca_{ij}^{T}\;(t)=0$ from the time *t* till the end of the simulation since calcium plays an important role in the function of the terminal.

### Neuroprotective Strategies

#### Levodopa Therapy

To alleviate PD symptoms, the most potent drug, L-DOPA, a precursor of DA, is typically administrated (Jankovic and Aguilar, 2008). During medication, serum L-DOPA is taken up from the blood into the extracellular fluid compartment by aromatic L-amino acid transporter by competing with other amino acids (Camargo et al., 2014; Figura et al., 2018). L-DOPA, thus absorbed into the bloodstream, later enters SNc terminals and gets converted to DA by aromatic L-amino acid decarboxylase (Khor and Hsu, 2007). In the proposed LIT model, serum L-DOPA uptake into the SNc terminal from the blood was modeled as a single step along with competition with other amino acids, such as tyrosine and tryptophan (Porenta and Riederer, 1982). It was described using the Michaelis-Menten equation (Chou, 1976) where serum L-DOPA competes with serum tyrosine and serum tryptophan for transporter (Reed et al., 2012) as given below:


(44) $$\begin{array}{l} V_{trans}=\frac{V_{trans}^{\max}*\left[LDOPA_{S}\right]}{K_{m}^{LDOPA_{s}}*\left(1+\frac{\left[TYR_{s}\right]}{K_{a}^{TYR_{s}}}+\frac{\left[TRP_{s}\right]}{K_{a}^{TRP_{s}}}\right)+\left[LDOPA_{S}\right]} \end{array}$$

where $V_{trans}^{\max}$ is the maximum flux through aromatic L-amino acid transporter, [*LDOPA*_*S*_] is the serum L-DOPA concentration, $K_{m}^{LDOPA_{s}}$ is the concentration of [*LDOPA*_*S*_] at which velocity of the transporter attained half of the maximal velocity, [*TYR*_*s*_] is the serum tyrosine concentration, and [*TRP*_*s*_] is the serum tryptophan concentration.${\;K}_{a}^{TYR_{s}}$ is the affinity constant for [*TYR*_*s*_], and $K_{a}^{TRP_{s\;}}$ is the affinity constant for [*TRP*_*s*_].

L-DOPA therapy was implemented in the proposed LIT model by the following criterion,


(45) $$\begin{array}{l} \left[LDOPA_{s}\right]\left(N_{sc}^{z},t\right)=\{\begin{array}{cc} 0, & N_{sc}^{z}\;(t)>T_{l}^{z}\\ \left[LDOPA_{s}^{med}\right], & N_{sc}^{z}\;(t)\leq T_{l}^{z} \end{array} \end{array}$$

(46) $$\begin{array}{l} T_{l}^{z}=P_{z}^{snc}-\left(pcl*P_{z}^{snc}\right) \end{array}$$

where $\left[LDOPA_{s}\right]\left(N_{sc}^{z},t\right)$ is the instantaneous serum [*LDOPA*] concentration based on the number of surviving SNc neurons or terminals at the time $\left(t\right)\left(N_{sc}^{z}\left(t\right)\right)$, $\left[LDOPA_{s}^{med}\right]$ is the serum [*LDOPA*] concentration during medication, $N_{sc}^{z}\;\left(t\right)$ is the instantaneous number of surviving SNc neurons or terminals, *pcl* is the percentage of SNc cell or terminal loss (25%) at which therapeutic intervention was employed (*pcl* = 0.25),${\;T}_{l}^{z}$ represents the number of surviving SNc cells or terminals at which therapeutic intervention was employed, and $P_{z}^{snc}$ is the population size of *z*(*z* = *soma or terminal*). In the present study, therapeutic intervention is given at 25% SNc cell or terminal loss.

#### SP Antagonist Therapy

It was reported that SP exacerbated dopaminergic neurodegeneration in mice (Wang et al., 2014), and, therefore, administrating SP antagonists creates neuroprotection of dopaminergic neurons in PD (Thornton and Vink, 2012, 2015; Johnson et al., 2017). In the proposed LIT model, SP antagonist effect was implemented as,


(47) $$\begin{array}{l} w_{spa}=w_{sp}*\delta_{spa} \end{array}$$

where *w*_*sp*_ is the influence of SP on *w*_*STN*→*SNc*_, δ_*spa*_ is the proportion of SP inhibition, and *w*_*spa*_ is the influence of SP on *w*_*STN*→*SNc*_ under SP antagonist therapy.

The SP antagonist therapy was implemented in the proposed LIT model by the following criterion,


(48) $$\begin{array}{l} \delta_{spa}\left(N_{sc}^{z},t\right)=\{\begin{array}{cc} 0, & N_{sc}^{z}\;(t)>T_{l}^{z}\\ \delta_{spa}^{med}, & N_{sc}^{z}\;(t)\leq T_{l}^{z} \end{array} \end{array}$$

where $\delta_{spa}\left(N_{sc}^{z},t\right)$ is the instantaneous proportion of SP inhibition based on the number of surviving SNc neurons or terminals at the time $\left(t\right)\left(N_{sc}^{z}\left(t\right)\right)$, $\delta_{spa}^{med}$ is the proportion of SP inhibition during therapy, $N_{sc}^{z}\;\left(t\right)$ is the instantaneous number of surviving SNc neurons or terminals, and ${\;T}_{l}^{z}$ represents the number of surviving SNc cells or terminals at which therapeutic intervention was employed (*z* = *soma or terminal*).

#### Glutathione Therapy

The impaired DA metabolism causes oxidative stress (ROS), leading to PD pathogenesis (Masato et al., 2019). It was reported that abnormal activity of vesicular monoamine transporter 2 (VMAT2) leads to reduced vesicular DA storage and increased cytoplasmic DA, which results in oxidative stress-induced degeneration of cell bodies (soma) and terminals (Kariya et al., 2005; Caudle et al., 2007; Pifl et al., 2014; Mingazov and Ugryumov, 2019). It was reported that glutathione (GSH) administration improves PD symptoms, but the underlying mechanism is unclear (Zeevalk et al., 2008; Hauser et al., 2009; Mischley et al., 2017). We suggest that glutathione administration might result in ROS scavenging, leading to neuroprotection (Li et al., 2015). In the proposed LIT model, the glutathione effect was implemented as,


(49) $$\begin{array}{l} \left[GSH_{gst}^{z}\right]=\left[GSH^{z}\right]+\left[GSH_{gs}^{z}\right] \end{array}$$

where $\left[GSH_{gs}^{z}\right]$ is the GSH concentration under glutathione therapy (*z* = *soma or terminal*), and [*GSH*^*z*^] is the GSH concentration.

The glutathione therapy was implemented in the proposed LIT model by the following criterion,


(50) $$\begin{array}{l} \left[GSH_{gs}^{z}\right]\left(N_{sc}^{z},t\right)=\{\begin{array}{cc} 0, & N_{sc}^{z}\;(t)>T_{l}^{z}\\ \left[GSH_{med}^{z}\right], & N_{sc}^{z}\;(t)\leq T_{l}^{z} \end{array} \end{array}$$

where $\left[GSH_{gs}^{z}\right]\left(N_{sc}^{z},t\right)$ is the instantaneous [*GSH*] therapy based on the number of surviving SNc neurons or terminals at the time $\left(t\right)\left(N_{sc}^{z}\left(t\right)\right)$, $N_{sc}^{z}\left(t\right)$ is the instantaneous number of surviving SNc neurons or terminals, $\left[GSH_{med}^{z}\right]$ is the [*GSH*] concentration dosage under *GSH* therapy, and ${\;T}_{l}^{z}$ represents the number of surviving SNc cells or terminals at which therapeutic intervention was employed (*z* = *soma or terminal*).

For statistical analysis, we have used the one-way ANOVA method to validate the significance of variance and rejected the null hypothesis when the *p*-value is <0.05 (Kim, 2017).

## Results

**Figure 2:**
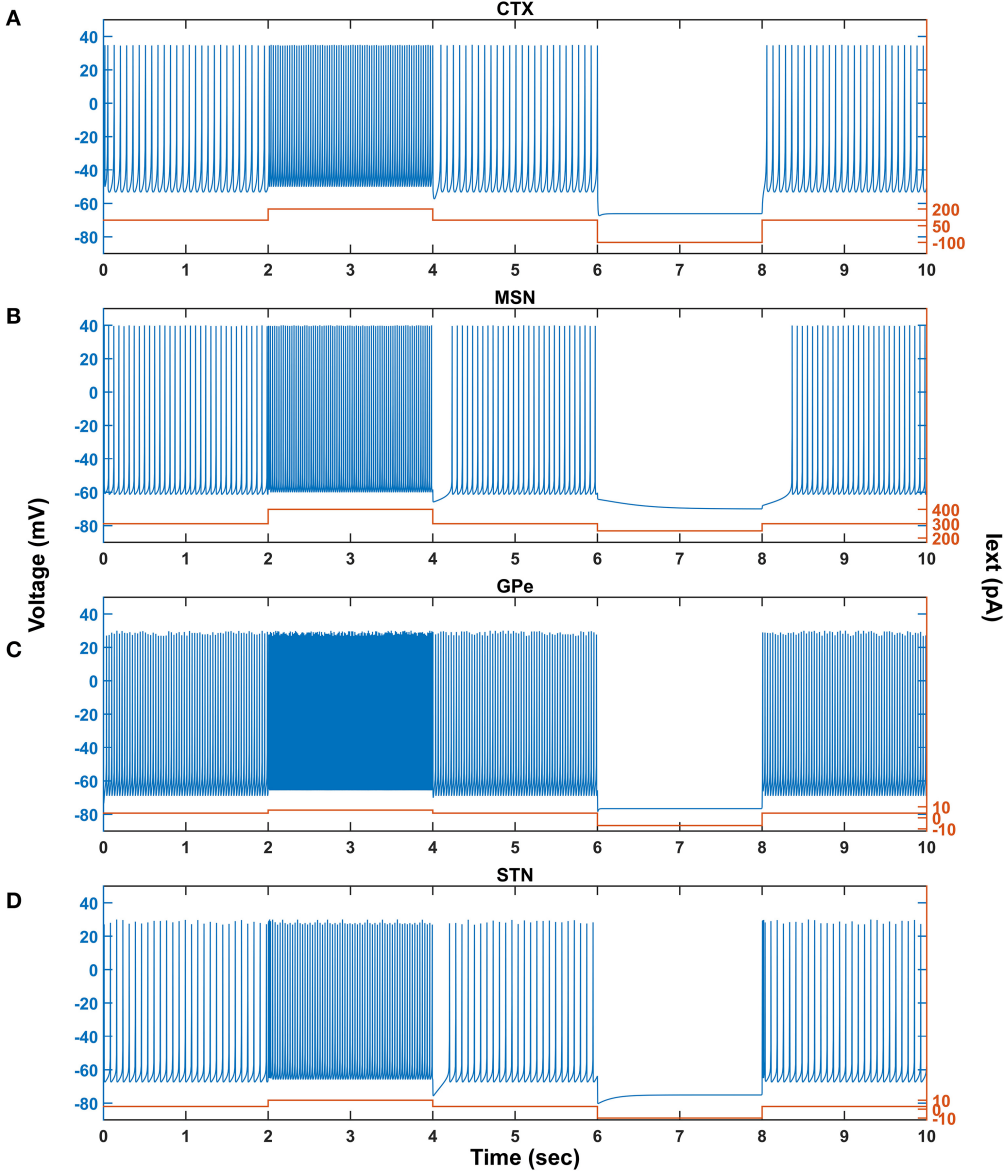
Characteristic behavior of individual neurons from a single isolated neuron. Characteristic behavior of individual CTX **(A)**, MSN **(B)**, GPe **(C)**, and STN **(D)** neuronal types. CTX, cortex; MSN, medium spiny neuron; GPe, globus pallidus externa; STN, subthalamic nucleus; Iext, external current; pA, picoampere; mV, millivolt; sec, second.

**Figure 3:**
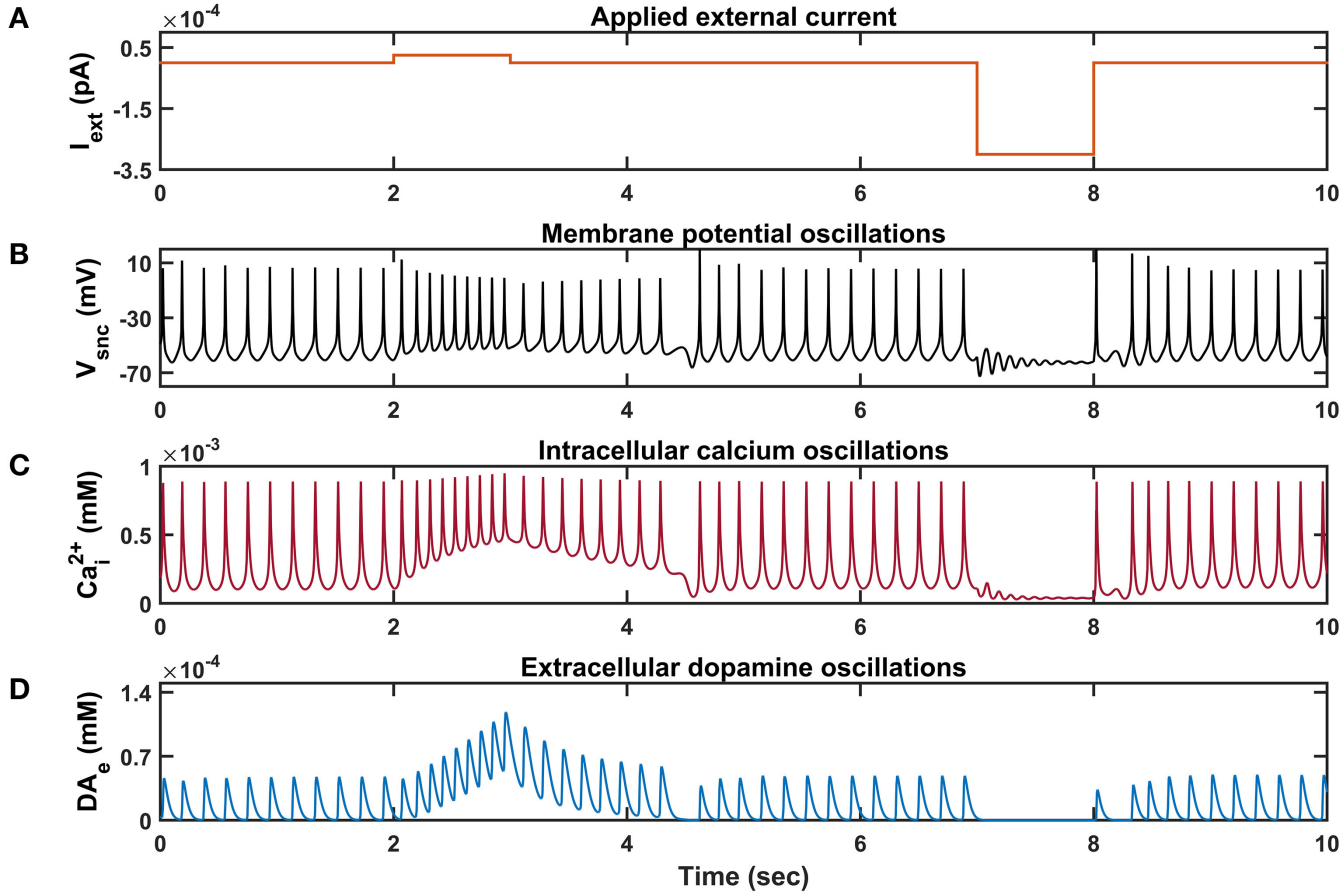
Characteristic behavior of individual SNc neuron simulated in isolation. **(A)** Applied external current (*I*_*ext*_), **(B)** membrane potential oscillations (*V*_*snc*_), **(C)** intracellular calcium oscillations $\left(Ca_{i}^{2+}\right)$, **(D)** extracellular dopamine concentration (*DA*_*e*_). pA, picoampere; mV, millivolt; sec, second; mM, millimolar.

**Figure 4:**
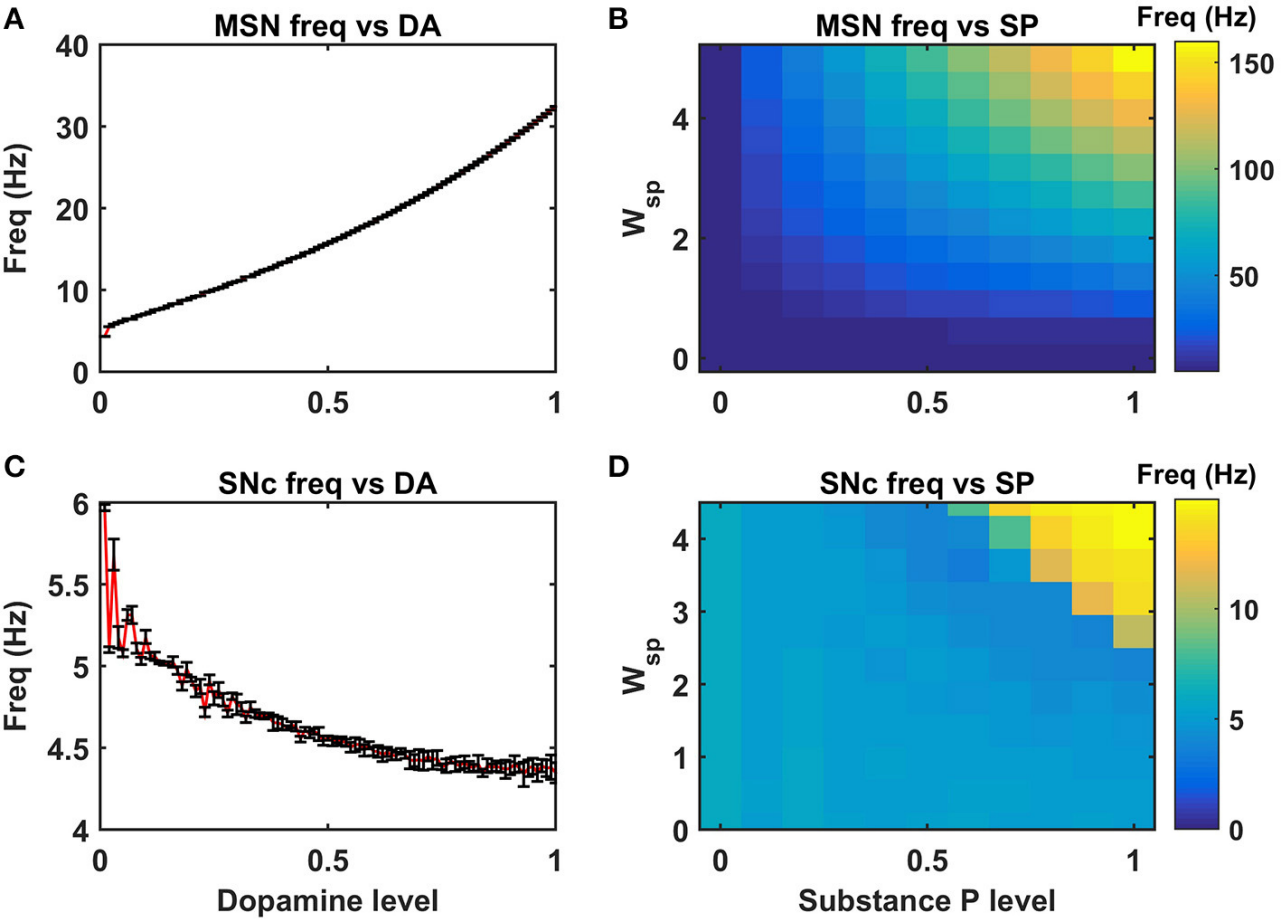
Neuromodulatory effects of DA and SP on MSN and SNc neuronal populations. Effect of dopamine on MSN **(A)** and SNc **(C)** populations. Effect of SP on MSN **(B)** and SNc **(D)**. MSN, medium spiny neuron; DA, dopamine; Freq, frequency; SP, substance P; SNc, substantia nigra pars compacta; *W*_*sp*_, scaling factor of SP influence; Hz, hertz.

We investigated the Izhikevich models of the neurons of CTX, MSN, GPe, and STN, which were chosen from the literature (Humphries et al., 2009; Michmizos and Nikita, 2011; Mandali et al., 2015) for their characteristic firing patterns and other biological properties (Figure 2). Along with the above Izhikevich neuronal models, we also investigated the biophysical neuronal model of SNc for its characteristic responses (Figure 3). Next, we explored the effect of DA and SP on the network of MSN and SNc neurons and compared them with published data (Figure 4).

**Figure 5:**
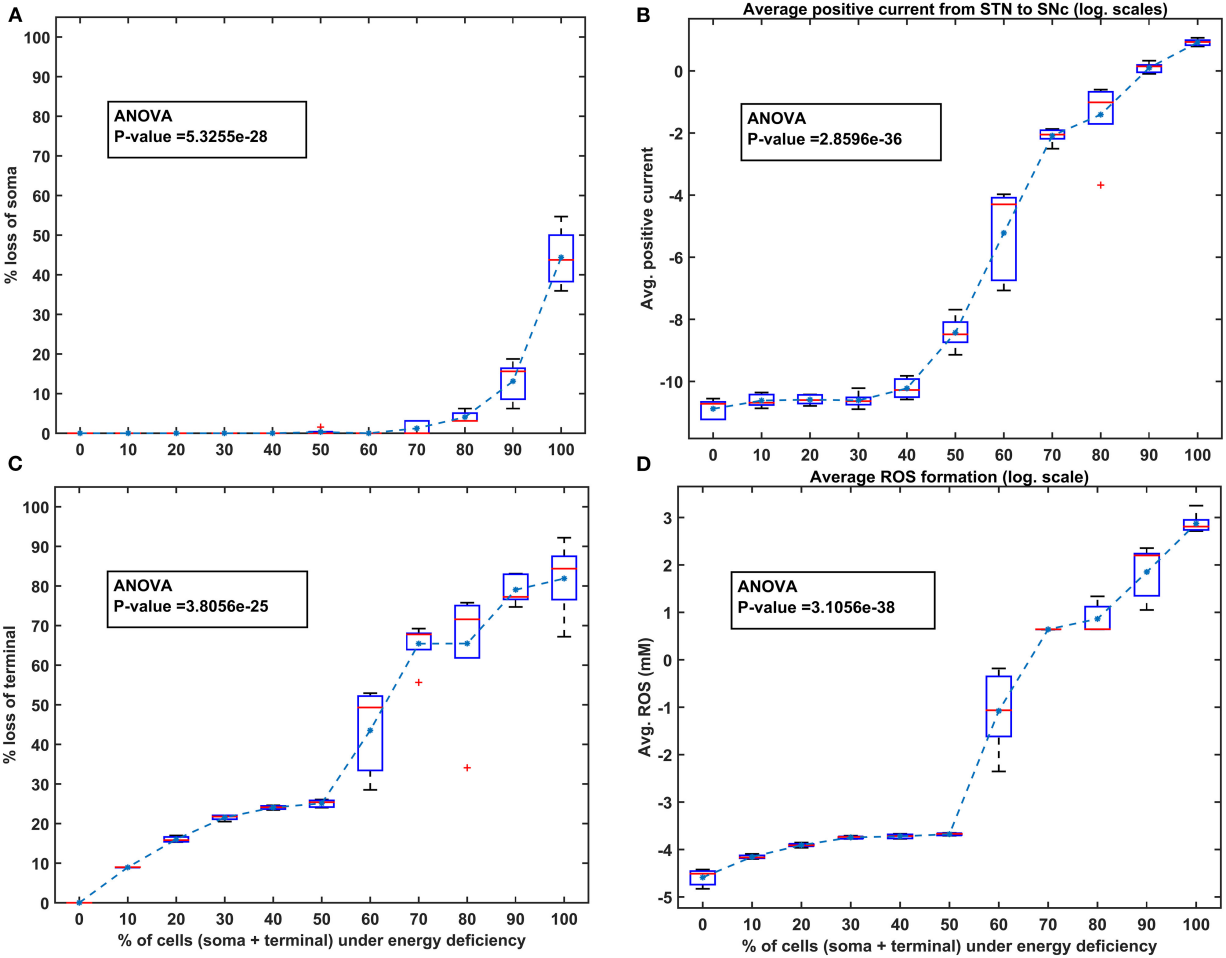
The model response under homogeneous energy deficiency. **(A)** The percentage loss of SNc somas, **(B)** the average positive current (log. scale) from STN to SNc, **(C)** the percentage loss of SNc terminals, **(D)** the average ROS concentration (log. scale). STN, subthalamic nucleus; SNc, substantia nigra pars compacta; ROS, reactive oxygen species; mM, millimolar.

**Figure 6:**
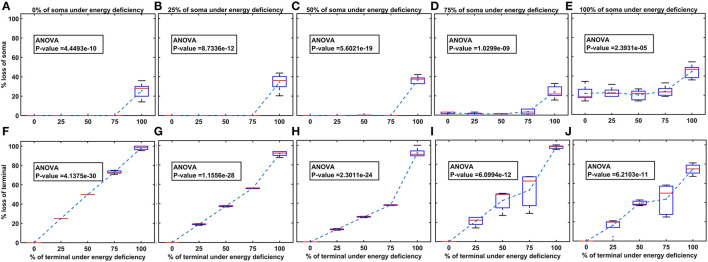
The model response under heterogeneous energy deficiency. **(A–E)** The percentage loss of SNc somas, **(F–J)** the percentage loss of SNc terminals. SNc, substantia nigra pars compacta.

**Figure 7:**
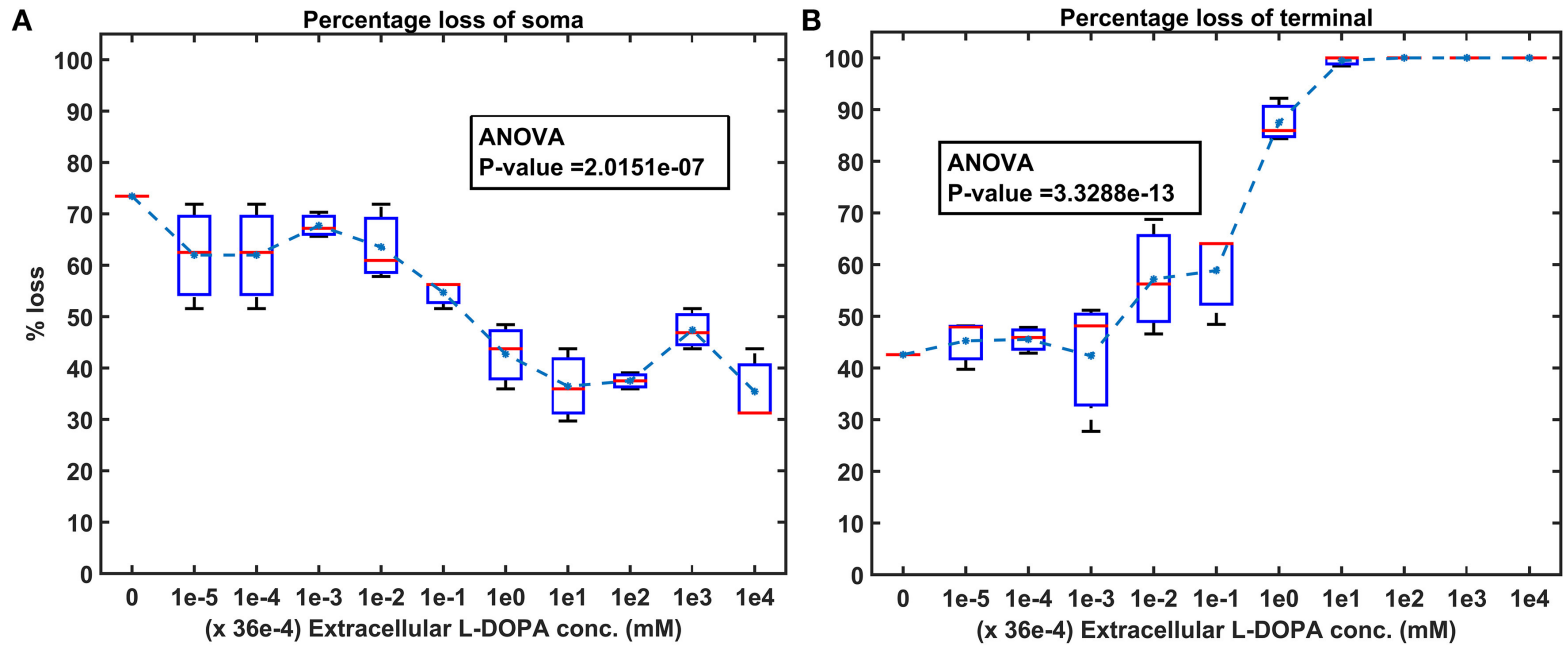
Model response to different extracellular L-DOPA under energy deficiency. **(A)** Percentage loss of somas for various extracellular L-DOPA concentrations, **(B)** percentage loss of terminals for various extracellular L-DOPA concentrations. conc, concentration; L-DOPA, levodopa; mM, millimolar.

**Figure 8:**
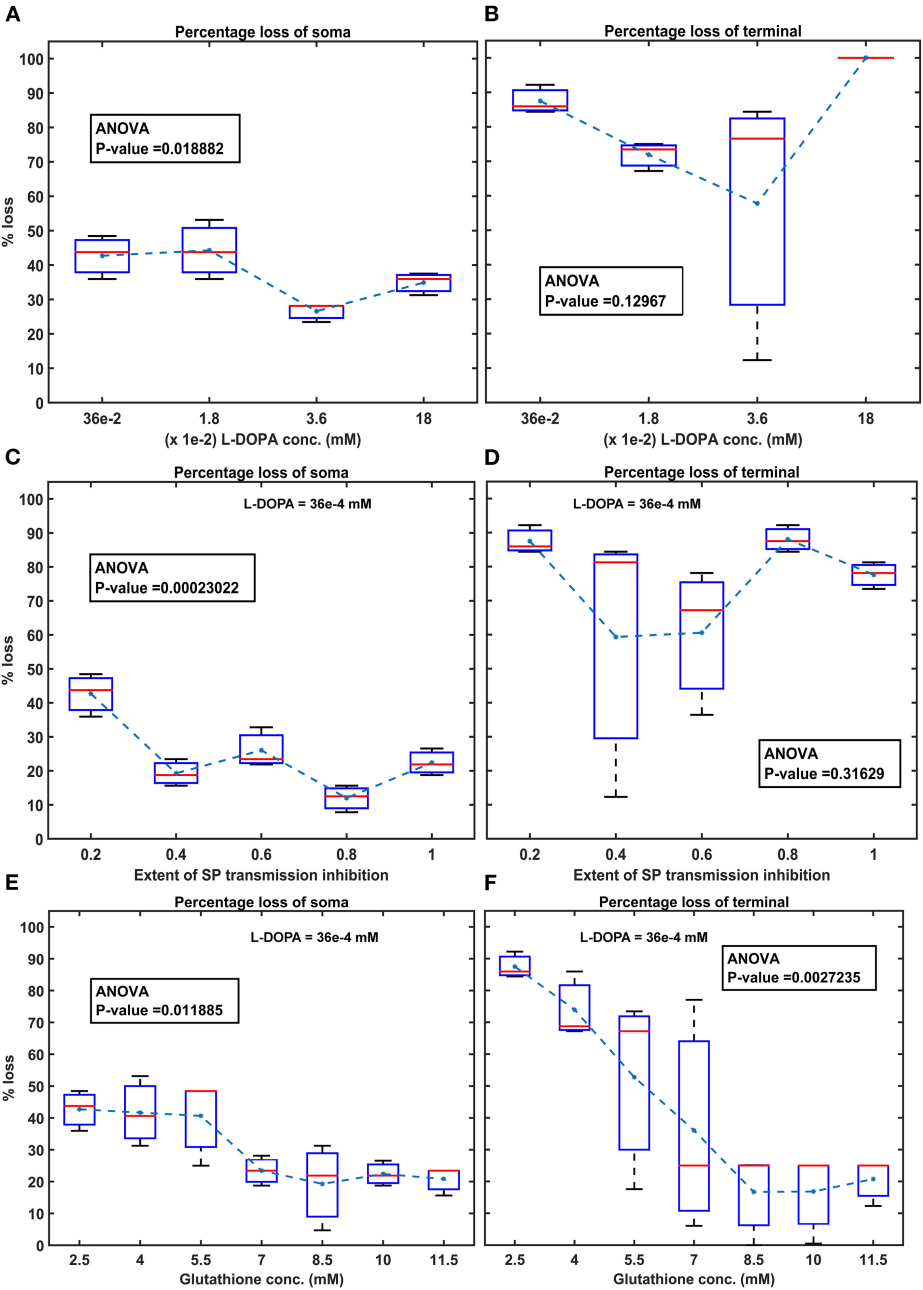
Model response to different therapeutics under energy deficiency. **(A)** Percentage loss of somas during L-DOPA therapy, **(B)** percentage loss of terminal during L-DOPA therapy, **(C)** percentage loss of somas during SP antagonist therapy, **(D)** percentage loss of terminals during SP antagonist therapy, **(E)** percentage loss of somas during glutathione therapy, (**F)** percentage loss of terminals during glutathione therapy. All the therapeutic interventions were initiated at 25% soma or terminal loss. conc, concentration; L-DOPA, levodopa; SP, substance P; mM, millimolar; ANOVA, analysis of variance.

Then, we show the effect of homogeneous (Figure 5) and heterogeneous (Figure 6) energy deficit conditions on the progression of SNc soma and terminal loss. Next, we show the effect of extracellular L-DOPA on the progression of SNc soma and terminal loss under energy deficit conditions (Figure 7). Finally, we explored various therapeutics, such as L-DOPA, SP antagonist, and glutathione (Figure 8), for their neuroprotective effect on the progression of SNc soma and terminal loss under energy deficit conditions.

### Characteristic Firing Response of Different Neuron Types

The response of a single neuron model of five different neuron types involved in the proposed LIT model for different external applied currents is shown in Figure 2. The basal firing frequency of the different neuronal types was matched with experimentally observed firing frequencies (Tripathy et al., 2014) by adjusting $I_{ij}^{x}$ parameter, which is given in Supplementary Material 2.

The combined (spontaneous and stimulus-driven) firing rate of the pyramidal cortical neuron in the model was tuned by adjusting $I_{ij}^{x}$ value, which falls in the range of 10 − 15 *Hz* (Figure 2A). Similarly, the combined (spontaneous and cortical-driven) firing rate of MSN was tuned such that it falls in the range of 10 − 20 *Hz*, which was observed experimentally (Mahon et al., 2006; Pitcher et al., 2007; Figure 2B).

The GPe neurons exhibit an atypical firing pattern where bursts and pauses appear aperiodically in a continuous tonic high-frequency firing (Kita and Kita, 2011; Hegeman et al., 2016). In the model, we adjusted $I_{ij}^{x}$ value such that the GPe spontaneous firing rate is ~ 30 *Hz*, which falls in the range of 8 − 60 *Hz* observed experimentally (Elias et al., 2008; Bugaysen et al., 2010; Lindahl et al., 2013; Figure 2C).

Unlike GPe neurons, STN neurons exhibit two distinct firing patterns experimentally: tonic pacemaking firing and phasic high-frequency bursting (Beurrier et al., 1999; Allers et al., 2003), and the STN neuronal model adapted here exhibits both types of firing patterns. In the model, we adjusted $I_{ij}^{x}$ value such that the STN spontaneous firing rate is ~ 13 *Hz*, which falls in the range of 6 − 30 *Hz* observed experimentally (Allers et al., 2003; Lindahl et al., 2013; Figure 2D).

Similar to STN neurons, SNc neurons experimentally exhibit two distinct firing patterns: background or low-frequency irregular tonic firing (3 − 8 *Hz*), and bursting or high-frequency regular phasic firing (~ 20 *Hz*) (Grace and Bunney, 1984a,b). In the model, SNc neurons spontaneously fire with a firing rate of ~ 4 *Hz* (Figure 3), which was observed experimentally, and the underlying calcium oscillation driving this spontaneous voltage oscillation was ranging in between ~ 1 *x* 10^−4^ *mM* and ~ 1 *x* 10^−3^ *mM*, and peaks to ~ 1 *x* 10^−3^ *mM* upon arrival of the action potential (Figure 3C; Dedman and Kaetzel, 1997; Ben-Jonathan and Hnasko, 2001; Wojda et al., 2008). This spontaneous calcium oscillation in the SNc terminal induces dopamine release, which was in the concentration range of (34 − 48) *x* 10^−6^ *mM* observed experimentally (Garris et al., 1997; Figure 3D). When depolarizing external current [continuous pulse $\left(I_{ext}=25\;x\;10^{-6}\;pA\right)$ and duration (1 *s*))] was injected, SNc neuron exhibited a bursting type of firing, which lasted for more than 1 s after the pulse was removed (Figure 2B, positive current), demonstrating the slow-adapting nature of SNc neuron due to an excess calcium build-up inside the neuron (Figure 3C, positive current; Kuznetsova et al., 2010). During the depolarizing current stimulation, SNc neurons that exhibit the property within a burst that spikes after an initial spike showed a decrease in amplitude (Figure 3B, positive current), which is a characteristic bursting property of SNc neurons (Grace and Bunney, 1984a). The dopamine concentration released by SNc neuron during depolarizing current stimulation peaked at ~ 118 *x* 10^−6^ *mM* (Figure 3D, positive current), which falls in the range of (90 − 220) *x* 10^−6^ *mM* observed experimentally (Chen and Budygin, 2007). Further increase in depolarizing current amplitude increases extracellular DA release exponentially but never exceeds beyond 1 *x* 10^−3^ *mM* (not shown; Gonon, 1988). When hyperpolarized external current [continuous pulse $\left(I_{ext}=-300\;x\;10^{-6}\;pA\right)$ and duration (1 *s*))] was injected, SNc neuron exhibited quiescent state until stimulation was removed (Figure 3B, negative current). Due to hyperpolarized current stimulation, the calcium oscillation in SNc neuron was minimal (Figure 3C, negative current), which resulted in the near absence of extracellular DA (Figure 3D, negative current).

The lateral connections in SNc, STN, and GPe neuronal populations were studied in the previous work (Muddapu et al., 2019). To simplify the proposed LIT model, no lateral connections were considered in CTX and MSN neuronal populations.

### Neuromodulatory Effect of Dopamine on MSN and SNc Neuronal Populations

DA affects both synaptic and intrinsic ion channels of MSN (Surmeier et al., 2007), and the combined (synaptic and intrinsic) effect of DA on MSN was formulated in the model. As the DA levels increase, the influence of cortical glutamatergic inputs on D1-type MSN increases, resulting in monotonously increasing firing frequency (Figure 4A), which was consistent with experimental (Cepeda et al., 1993) and previous modeling studies (Humphries et al., 2009). In addition, SP also affects synaptic ion channels of MSN, especially glutamatergic afferents from cortical neurons (Blomeley and Bracci, 2008; Blomeley et al., 2009). As the SP levels [or SP scaling factor (*W*_*sp*_))] increase, the influence of cortical glutamatergic inputs on D1-type MSN increases, resulting in monotonously increasing firing frequency (Figure 4B), which was similar to experimental (Blomeley and Bracci, 2008) and other modeling studies (Buxton et al., 2017).

DA affects both synaptic ion channels at a single neuronal level and lateral connections (Muddapu et al., 2019) at the network level of SNc neurons. As the DA level increases, the influence of synaptic and lateral connection inputs on SNc increases, resulting in monotonously decreasing firing frequency (Figure 4C), which was similar to experimental (Hebb and Robertson, 1999; Vandecasteele et al., 2005; Tepper and Lee, 2007; Ford, 2014) and other modeling studies (Muddapu et al., 2019). In addition, SP also affects the synaptic ion channels of SNc, especially glutamatergic afferents from STN (Brimblecombe and Cragg, 2015; Thornton and Vink, 2015). As the SP level [or SP scaling factor (*W*_*sp*_))] increases, the influence of STN glutamatergic inputs on SNc increases, resulting in monotonously increasing firing frequency (Figure 4D), which was similar to experimental studies (Brimblecombe and Cragg, 2015). The detailed analysis of DA effect on STN and GPe neuronal populations was described in the previous work (Muddapu et al., 2019).

### Energy Deficiency Occurring Similarly in SNc Somas and Terminals

To investigate energy deficiency as the possible root cause of SNc cell loss in PD, we simulated ischemic conditions by modulating glucose and oxygen inputs to the model. The ischemic condition was implemented in two scenarios, as SNc somas (in the midbrain) and terminals (in the striatum) are located far from each other: homogeneous (energy deficiency occurs similarly in somas and terminals) and heterogeneous (energy deficiency occurs differently in somas and terminals). Homogeneous energy deficiency was implemented by reducing glucose and oxygen values by the same proportions in both SNc somas and terminals. The homogeneous energy deficiency causes soma loss at 70% energy deficiency (Figure 5A). The soma loss at high energy deficiency can result from the threshold-like effect of STN on SNc. The influence of STN on SNc was observed by monitoring currents from STN to SNc, which showed higher positive currents after 50% of somas and terminals were in energy deficiency (Figure 5B). However, soma loss does not occur until 70% homogeneous energy deficiency. So, a threshold-like phenomenon exists between STN and SNc, after which the runaway effect kicks in. Contrarily, terminal loss starts with just 10% of somas and terminals in energy deficiency (Figure 5C). The terminal loss at low energy deficiency can result from a ROS build-up due to energy deficiency. As a result, increased ROS production in SNc terminals was observed from 10% homogeneous energy deficiency, leading to terminal degeneration (Figure 5D).

### Energy Deficiency Occurring Differently in SNc Somas and Terminals

Heterogeneous energy deficiency was implemented by reducing glucose and oxygen values by different proportions in SNc somas and terminals. The heterogeneous energy deficiency causes soma loss at only 100% energy deficiency in terminals when the energy deficiency in somas is set at 0%, 25%, or 50% (Figures 6A–C). When 75% of somas were energy deficient, significant loss of soma was observed when 100% of terminals are in energy deficiency (Figure 6D). However, when 100% of somas were in energy deficiency, significant loss of soma was observed at all percentages of energy deficiency in terminals, and maximum loss of soma (~ 45%) was observed when 100% of terminals were in energy-deficient condition (Figure 6E). Contrarily, the terminal loss was observed for all non-zero percentages of energy deficiency in somas and terminals (Figures 6F–J). The terminal loss increases with an increase in the percentage of terminals in energy deficiency for all percentages of somas in energy deficiency (Figures 6F–J).

### Effect of Extracellular L-DOPA

To investigate the effect of extracellular (serum) L-DOPA on SNc somas and terminal loss under energy deficiency (100% energy deficiency), we have modified extracellular L-DOPA concentration in the range from 36 *x* 10^−9^ *mM* to 36 *mM* in multiples of 10 (Figure 7A). At an extracellular L-DOPA concentration of zero, the percentage loss of somas and terminals was ~ 75% and ~ 40%, respectively, under 100% homogeneous energy deficiency. At lower concentrations of extracellular L-DOPA, ranging from 36 *x* 10^−9^ *mM* to 36 *x* 10^−6^ *mM*, a more significant loss (average value of ~ 63%) (Figure 7A) of SNc somas was observed when compared to SNc terminals (average value of ~ 46%) (Figure 7B). Contrarily, at higher concentrations of extracellular L-DOPA, ranging from 36 *x* 10^−4^ *mM* to 36 *mM*, more of SNc terminal loss (average value of ~ 95%) (Figure 7B) was observed when compared to SNc somas (average value of ~ 37%) (Figure 7A). At extracellular L-DOPA concentration of 36 *x* 10^−5^ *mM*, the percentage loss of SNc somas and terminals was similar, which was in the range of 50 − 60%, and this value of the extracellular L-DOPA concentration was observed in previous studies (Khor and Hsu, 2007; Reed et al., 2012; Cullen and Wong-Lin, 2015).

### L-DOPA and Its Adjuvant Therapies

To test the hypothesis of L-DOPA-induced toxicity, we have administered a range of external L-DOPA concentrations in the model when the percentage loss of somas or terminals crosses was 25% due to energy deficiency. When external L-DOPA concentration (36 *x* 10^−5^ *mM*) administered was near the basal value (basal L-DOPA concentration was fixed at 36 *x* 10^−5^ *mM*), it was observed that the percentage loss of SNc somas and terminals was not altered much. When external L-DOPA concentration administered was in the range from 36 *x* 10^−4^ *mM* to 36 *x* 10^−3^ *mM*, it was observed that the percentage loss of SNc somas was decreasing; however, the percentage loss of SNc terminals was not significantly altered. On the contrary, when administered external L-DOPA concentration was above 36 *x* 10^−3^ *mM*, it was observed that the percentage loss of SNc somas and SNc terminals increased (Figures 8A,B).

The simulation results showed that L-DOPA, indeed, induced toxicity in SNc cells at higher concentrations, which might be due to excitotoxicity or oxidative stress, or both. To evade L-DOPA toxicity in all stages of L-DOPA therapy in the case of PD, we need to understand the mechanism behind the toxicity. To do so, we co-administered two different drugs along with L-DOPA, namely, SP antagonist and glutathione (ROS scavenger), which targets overexcitation in SNc somas (by reducing SP-mediated excitatory inputs to SNc) and an ROS build-up in SNc terminals (by scavenging ROS), respectively. When SP antagonists are co-administered (with administered L-DOPA concentration fixed at 36 *x* 10^−4^ *mM*), it was observed that the percentage loss of SNc somas was decreasing with increasing inhibition of SP transmission (Figure 8C). However, there was no significant change in the percentage loss of SNc terminals across the different extents of SP transmission inhibition (Figure 8D). When glutathione was co-administered (with administered L-DOPA concentration fixed at 36 *x* 10^−4^ *mM*), it was observed that the percentage loss of SNc somas and terminals was decreasing with increasing glutathione concentration (Figures 8E,F).

## Discussion

### L-DOPA-Induced Toxicity Model

#### Site of Degeneration

This computational study aims to develop a model of SNc-striatum, which helps us understand L-DOPA-induced toxicity in SNc neurons under energy deficiency conditions. From both homogeneous and heterogeneous energy deficiency results, it suggests that SNc (axonal) terminals are more vulnerable to energy imbalance when compared to SNc cell bodies (somas), which was also observed experimentally, when an injury is initiated at axonal terminals (Burré et al., 2010; Cheng et al., 2010; Giguère et al., 2019; Wong et al., 2019). The higher positive currents from STN projections to SNc might lead to excitotoxic loss of SNc somas (Figure 5A), and increased ROS production might lead to increased SNc terminal loss (Figure 5C). DA transporters, which play a crucial role in DA neurotransmission, were depleted more in axonal terminals compared to cell bodies in early PD (Fazio et al., 2018). From these studies, it can be postulated that pathogenesis starts at axonal terminals, which are more vulnerable to energy deficiencies and, therefore, are ideal sites for developing novel disease-modifying therapeutics.

#### Significance of Basal Extracellular L-DOPA

The loss of SNc somas was more when compared to SNc terminals at lower concentrations of extracellular L-DOPA (Figure 7). This might be due to lower extracellular DA levels as a result of lower extracellular L-DOPA concentrations and lower vesicular DA levels (due to reduced packing of DA into vesicles as a result of lower energy levels), causing disinhibition of SNc somas (as a result of lesser cortical excitation of MSNs), which are already in a low energy state. Due to disinhibition and energy deficiency, SNc somas might become overactive, which leads to a calcium build-up, resulting in excitotoxic loss of SNc somas (Albin and Greenamyre, 1992; Muddapu et al., 2019).

Contrarily, the loss of SNc terminals was more when compared to SNc somas at higher concentrations of extracellular L-DOPA (Figure 7). This might be due to higher cytoplasmic DA levels as a result of higher extracellular L-DOPA concentrations, lower vesicular packaging of DA (due to lower energy levels), and L-DOPA-induced stimulation of DA metabolism (Mosharov et al., 2009), resulting in DA-mediated oxidative stress in the SNc terminals (Farooqui, 2012; Morrison et al., 2012). Due to higher DA levels and energy deficiency, DA in SNc terminals causes oxidative stress, resulting in SNc terminal loss. At higher concentrations of extracellular L-DOPA, loss of SNc somas was lower compared to lower concentrations of extracellular L-DOPA as a result of the restoration of inhibitory tone from MSNs due to higher extracellular DA concentrations. The extracellular L-DOPA concentration of 36 *x* 10^−5^ *mM* was considered as basal extracellular L-DOPA concentrations in the proposed LIT model. At these values, the percentage loss of SNc somas and terminals was similar, which was observed in previous studies (Khor and Hsu, 2007; Reed et al., 2012; Cullen and Wong-Lin, 2015). Our model was able to show the significance of basal extracellular L-DOPA concentrations, which is needed to be maintained for normal functioning.

#### Adjuvant Therapies

When external L-DOPA concentration administered was in the range from 36 *x* 10^−4^ *mM* to 36 *x* 10^−3^ *mM*, it was observed that the percentage loss of SNc somas was decreasing, suggesting the neuroprotective benefits of L-DOPA therapy in altering or halting the progression of the SNc cell loss. However, this neuroprotective effect was not seen in the case of SNc terminals. When external L-DOPA concentration administered was above this range, the neuroprotective effect of L-DOPA therapy diminished in the case of SNc somas.

To prevent L-DOPA-induced toxicity, two different adjuvant therapies were carried on. In the first scenario, SP antagonist was co-administrated along with L-DOPA, which resulted in a further decrease in SNc soma loss, but no significant change in SNc terminal loss. From this, we can state that inhibiting excitotoxicity in SNc somas does not decrease SNc terminal loss, which suggests that excitotoxicity in SNc somas does not contribute to oxidative stress in SNc terminals in L-DOPA-induced toxicity. In the second scenario, glutathione was co-administrated along with L-DOPA, which decreased both SNc soma and terminal loss. From this, we can state that inhibiting oxidative stress in SNc terminals did reduce the loss of SNc somas, which suggests that oxidative stress in SNc terminals does contribute to excitotoxicity in SNc somas in L-DOPA-induced toxicity. From these results, we can suggest that adjunct therapies, such as antioxidants (Pardo et al., 1993, 1995; Walkinshaw and Waters, 1995; Carvey et al., 1997; Borah and Mohanakumar, 2010; Betharia et al., 2019; Nikolova et al., 2019; Deng et al., 2020), and other potential therapies, such as D2 agonists (Asanuma et al., 2003), glycogen synthase kinase 3 inhibitors (Choi and Koh, 2018), and calcium-binding protein drugs (Isaacs et al., 1997), co-administrated along with L-DOPA, should be able to evade L-DOPA toxicity in all stages of PD.

#### Insights Into the Mechanism of L-DOPA-Induced Toxicity

The simulation results showed that the L-DOPA-induced toxicity in cell bodies and axonal terminals of SNc neurons was autoxidation irrelevant and autoxidation relevant, respectively. In the case of cell bodies, excess DA in the striatum due to L-DOPA therapy stimulates glutamatergic cortical inputs to MSNs, which leads to overexcitation of MSNs. The overexcited MSNs co-release SP along with GABA onto SNc neurons. SP modulates SNc glutamatergic inputs in such a way that it overexcites SNc neurons, resulting in excitotoxic neuronal loss in SNc. However, in the case of axonal terminals, excess DA in terminals due to L-DOPA therapy leads to autooxidation of DA. The autoxidation of DA results in the production of free radicals, which lead to oxidative stress in SNc axonal terminals, resulting in axonal synaptic pruning of SNc neurons. The study suggests that L-DOPA-induced toxicity occurs by two mechanisms: DA-mediated oxidative stress in axonal terminals of SNc neurons and by exacerbating STN-mediated overexcitation in cell bodies of SNc neurons.

To summarize the main outcome of the present modeling study:

- SNc (axonal) terminals are more vulnerable to energy deficiency than SNc somas.
- Basal extracellular L-DOPA concentration is needed to maintain for normal functioning of the neuron.
- Adjuvant therapies, along with L-DOPA, such as glutathione, result in evading L-DOPA-induced toxicity.
- L-DOPA-induced toxicity in cell bodies and axonal terminals of SNc neurons was autoxidation irrelevant and autoxidation relevant, respectively.

### SNc Positive Feedback Loops—Scope of Vulnerability

#### Normal Scenario

In normal conditions, there is no SNc cell or terminal loss where SNc maintains the dopaminergic tone on its target regions, such as STN, D1-MSN(G), and D1-MSN(GS). In the first loop (Figure 8), normal dopaminergic tone to D1-MSN(G) results in inhibition of SNc by GABAergic projections. In the second loop (Figure 8), normal dopaminergic tone to D1-MSN(GS) results in inhibition of SNc by GABA and lesser excitation of SNc by SP due to DA-SP feedback (Brimblecombe and Cragg, 2015; Thornton and Vink, 2015). In the third loop (Figure 8), normal dopaminergic tone to STN results in lesser excitation of SNc by glutamatergic projections (Hassani et al., 1997; Magill et al., 2001; Yang et al., 2016).

#### Pathological Scenario

Under pathological conditions, there is an SNc cell or terminal loss where SNc fails to maintain the dopaminergic tone in its target regions, such as STN, D1-MSN(G), and D1-MSN(GS). In the first loop (Figure 8), DA deficiency in the striatum causes lesser excitation of D1-MSN(G) by the cortex, which, by feedback, results in disinhibition of SNc. In other words, initial DA deficiency due to SNc cell loss causes lesser excitation of D1-MSN(G), which disinhibits SNc, resulting in further SNc cell loss due to excitotoxicity, which acts as positive feedback. In the second loop, DA deficiency in the striatum causes lesser excitation of D1-MSN(GS) by the cortex, which results in disinhibition (through GABA) and further excitation of SNc (through SP, due to low DA, the effect of SP gets enhanced). Thus, the disinhibition of SNc happens in a manner similar to the first loop; however, the overexcitation of SNc happens due to the DA-SP feedback mechanism, which also acts as positive feedback. In the third loop, DA deficiency causes overexcitation of STN, which results in the overactivation of SNc. In other words, initial DA deficiency due to SNc cell loss causes overexcitation of STN, which, in turn, overexcites SNc by a positive feedback mechanism, resulting in further SNc cell loss due to excitotoxicity.

#### Medication Scenario—Optimal L-DOPA Dosage

In medication conditions, L-DOPA is administrated where dopaminergic tone to SNc target regions [STN, D1-MSN(G), D1-MSN(GS)] is restored. If the administrated L-DOPA dosage goes beyond a certain threshold, overexcited D1-MSN(GS) through the DA-SP feedback mechanism makes SNc hyperactive, resulting in SNc cell loss due to excitotoxicity. Along with SNc cell body loss, SNc terminals also undergo degeneration due to excess DA-causing oxidative stress. To summarize, L-DOPA-induced toxicity in SNc does not occur when L-DOPA dosage is below the threshold, which results in the survival of remaining SNc cells. However, if L-DOPA dosage goes beyond a threshold, from that point onward, the aforementioned runaway effect kicks in, leading to a progressive and irrevocable cell loss in SNc. Thus, it is evident that L-DOPA might be toxic to SNc neurons under high dosage, which triggers a runaway effect, resulting in uncontrollable SNc cell loss.

**Figure 9:**
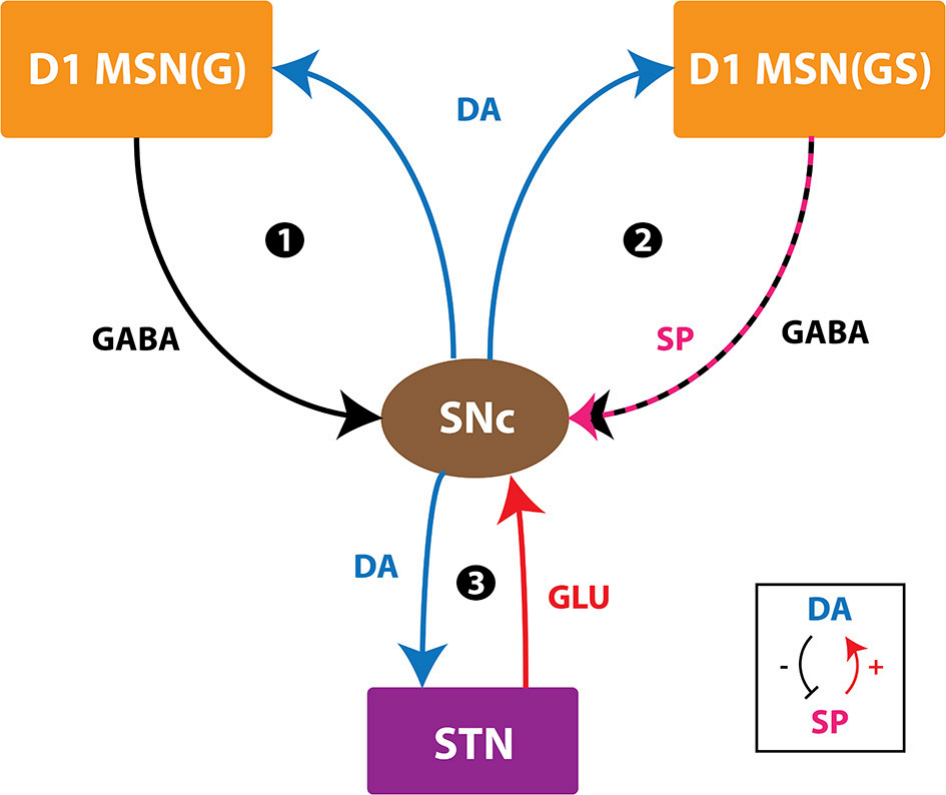
Positive feedback loops of SNc. SNc, substantia nigra pars compacta; STN, subthalamic nucleus; D1 MSN(G), D1-type DA receptor-expressing medium spiny neuron (release GABA); D1 MSN(GS), D1-type DA receptor-expressing medium spiny neuron (co-release GABA and SP); DA, dopamine; SP, substance P; GLU, glutamate; GABA, gamma-aminobutyric acid. Inset, DA-SP feedback.

In Section Effect of Extracellular L-DOPA, we studied the effect of L-DOPA on the survivability of SNc somas and terminals under energy deficiency. Simulations have shown that the basal level of extracellular L-DOPA is required for the normal functioning of cellular processes within SNc cells. If the concentration levels go below or above this basal level, all three loops of Figure 9 tend to operate in the pathological state, which eventually leads to SNc cell loss (Figure 7). In Section L-DOPA and Its Adjuvant Therapies, we studied the ability of L-DOPA therapy to alter the progression of SNc cell loss (soma and terminal) under energy deficiency. Simulations show that there exists a twilight L-DOPA dosage at which SNc cell loss was minimal (Figures 8A,B). At this L-DOPA dosage, all three loops in Figure 9 tend to operate in an optimal state where pathological influence from all three loops on SNc cells was minimal. If the dosage deviates from this optimal regime, all three loops (Figure 9) tend to operate in a pathological state. In order words, when L-DOPA dosage is very low, the overall effect (overexcitation leading to runaway effect) on SNc cells will lead to their degeneration due to excitotoxicity as a result of disinhibition (through the first loop), disinhibition, overexcitation (through the second loop), and overexcitation (through the third loop). When L-DOPA dosage is very high, its overall effect on SNc cells will eventually lead to their degeneration due to excitotoxicity: excess L-DOPA causes DA-induced oxidative stress in SNc cells, leading to loss of dopaminergic tone in SNc target nuclei. It is shown that as external L-DOPA is administered, the percentage loss of SNc somas comes down as the L-DOPA concentration increases till 36 *x* 10^−3^ *mM*; concentration levels beyond 36 *x* 10^−3^ *mM* result in diminishing neuroprotective effect of L-DOPA (Figure 8A).

#### Medication Scenario—Role of Adjuvant Drugs

We hypothesize that L-DOPA-induced toxicity in SNc cells at higher L-DOPA concentrations might be caused by excitotoxicity, oxidative stress, or both. To investigate this hypothesis and evade L-DOPA toxicity in all stages of L-DOPA therapy in PD, we need to understand the mechanism behind the toxicity. To study it *in silico*, we co-administered two different drugs along with L-DOPA - SP antagonists and glutathione. When SP antagonists were co-administered, it was observed that the percentage loss of SNc somas was decreasing with increasing inhibition of SP transmission (SP antagonist reduces SP-induced excitation in SNc; Figure 8C). However, there was no significant change in the percentage loss of SNc terminals (Figure 8D). From this, we can say that excitotoxicity in SNc soma was reduced when SP antagonist is co-administrated along with L-DOPA, which means L-DOPA-induced toxicity in SNc soma was occurring due to excitotoxicity. When glutathione was co-administered along with L-DOPA, the percentage loss of SNc somas and terminals was observed to decrease with increasing glutathione concentration (Figures 8E,F). From this, we can say that oxidative stress in SNc terminals was reduced when glutathione was administrated along with L-DOPA but not with SP antagonist. This means that L-DOPA-induced toxicity in SNc terminals was occurring due to oxidative stress. In addition, glutathione co-administrated with L-DOPA increases the survivability of SNc somas, which means oxidative stress in SNc terminals does contribute to excitotoxicity in SNc somas in L-DOPA-induced toxicity.

To summarize the main interpretation of the present modeling study:

- SNc neurons are involved in three positive feedback loops.
- Under energy deficiency, these positive feedback loops exacerbate the vulnerability of SNc neurons.
- Under L-DOPA medication, if the dosage goes beyond a threshold, which triggers a runaway effect, resulting in uncontrollable SNc cell loss.
- At ideal L-DOPA dosage, all positive feedback loops operate in an optimal state where pathological influence from all loops on SNc cells was minimal.
- Oxidative stress in SNc terminals does contribute to excitotoxicity in SNc somas in L-DOPA-induced toxicity. Contrarily, excitotoxicity in SNc somas does not contribute to oxidative stress in SNc terminals in L-DOPA-induced toxicity.

### Limitations and Future Directions

Although the proposed model captures the exciting results of L-DOPA-induced toxicity, it is not without limitations. For example, in the proposed model, the serotonergic system was not considered, which also takes up L-DOPA and contributes to striatal DA levels (Stansley and Yamamoto, 2015; Svenningsson et al., 2015). This DA release from serotonergic terminals can even contribute to L-DOPA-induced dyskinesias (Carta et al., 2008; Carta and Tronci, 2014). Similarly, interneurons in the striatum were also not considered for simplifying the model. In the proposed model, we have considered the DA modulation on the neural activity as an immediate effect rather than a slow process. However, the effect of neuropeptide on neural activity was delayed by 40 *ms* based on previous studies (Buxton et al., 2017). In future modeling studies, we would like to incorporate the delayed DA modulation on the neural activity, which provides a more realistic effect of neuromodulators.

The ischemic condition was implemented in the proposed model by lowering glucose and oxygen levels, which can be extended by adding a blood vessel module (Cloutier et al., 2009) and varying cerebral blood flow to simulate ischemia condition more realistically. In the proposed model, stress was monitored in SNc neurons alone, which can be extended to other neuronal types in the model by monitoring stress levels, where an intracellular calcium build-up can be a stress indicator (Bano and Ankarcrona, 2018). To do so, all neuronal types should be modeled as conductance-based models where calcium dynamics should be included. Our studies show that L-DOPA dosage plays an important role in the progression of the disease. The proposed model will be integrated with a behavioral model of cortico-basal ganglia circuitry (Muralidharan et al., 2018; Nair et al., 2022a) to show the effect of L-DOPA-induced toxicity at the behavioral level and optimize the L-DOPA dosage to achieve maximum effect on the symptoms with a minimal dosage of the drug (Nair et al., 2022b).

We suggest some experimental approaches to validate some of the predictions from our modeling study. Under induced progressive energy deficiency conditions in animal models (Puginier et al., 2019), L-DOPA administration at moderate levels can also be toxic, which needs to be studied by measuring metabolites of the DA autoxidation process. To study the effects of L-DOPA-induced toxicity in SNc somas in midbrain and SNc terminals in the striatum, similar toxin-induced animal models can be used, where oxidative stress in terminals can be examined by monitoring the levels of free radicals, and excitotoxicity in somas can be examined by monitoring calcium levels (Wong et al., 2019). By co-administering antioxidants along with L-DOPA in toxin-induced animal models (Pardo et al., 1993, 1995; Walkinshaw and Waters, 1995; Carvey et al., 1997; Borah and Mohanakumar, 2010; Betharia et al., 2019; Nikolova et al., 2019), the progression of SNc soma and terminal loss can be altered along with prolonging the “honeymoon period” of L-DOPA therapy (Holford and Nutt, 2008; Stocchi et al., 2010; Erro et al., 2016).

## Conclusion

In conclusion, we believe that the proposed model provides significant insights into understanding the mechanisms behind L-DOPA-induced toxicity under energy deficiency conditions. From simulation results, it was shown that SNc terminals are more vulnerable to energy imbalances when compared to SNc somas. The study suggests that L-DOPA-induced toxicity occurs differently in SNc somas and terminals; in the case of SNc somas, it might be due to excitotoxicity caused by STN, and, in the case of SNc terminals, it might be due to oxidative stress caused by dopamine autoxidation. From adjuvant therapies, it was clear that co-administering antioxidants, along with L-DOPA, can be neuroprotective. Based on the aforementioned modeling efforts and some earlier ones (Muddapu et al., 2019), we are trying to understand the root cause of PD neurodegeneration as energy deficiency occurring at different neural hierarchies: subcellular, cellular, and network levels.

## Data Availability Statement

The datasets presented in this study can be found in online repositories. The names of the repository/repositories and accession number(s) can be found at: http://modeldb.yale.edu/263719 - accession: patrick.

## Author Contributions

VM contributed to conceptualization, model development, data curation, formal analysis, investigation, methodology, and manuscript writing. VC contributed to conceptualization, model development, data curation, formal analysis, investigation, methodology, manuscript writing, and supervision. KV and KR contributed to conceptualization and model development. All authors contributed to the article and approved the submitted version.

## Conflict of Interest

The authors declare that the research was conducted in the absence of any commercial or financial relationships that could be construed as a potential conflict of interest.

## Publisher's Note

All claims expressed in this article are solely those of the authors and do not necessarily represent those of their affiliated organizations, or those of the publisher, the editors and the reviewers. Any product that may be evaluated in this article, or claim that may be made by its manufacturer, is not guaranteed or endorsed by the publisher.
