# Supplementary material for: A Multi-Scale Computational Model of Levodopa-Induced Toxicity in Parkinson's Disease
Source: Front Neurosci. 2022 Apr 19;16:797127. doi: 10.3389/fnins.2022.797127 (PMC9063169; doi:10.3389/fnins.2022.797127)
Supplement: Supplementary file 1 [file Data_Sheet_1.PDF]

SUPPLEMENTARY MATERIAL

A Multi-Scale Computational Model of Levodopa-Induced Toxicity in Parkinson's disease

Vignayanandam Ravindernath-Jayashree Muddapu[1], Karthik Vijayakumar[2], Keerthiga Ramakrishnan[2], V Srinivasa Chakravarthy[1, #]

Corresponding Author: Prof V. Srinivasa Chakravarthy (schakra@ee.iitm.ac.in)

S1: Different Population Sizes

Table S1: Different population sizes in the proposed LIT model (Oorschot, 1996).

Network type	Size (# of nodes)
SNc (soma)	$8\ x\ 8\ (64)$
SNc (terminal)	$32\ x\ 32\ (1024)$
D1-MSN (G)	$32\ x\ 32\ (1024)$
D1-MSN (GS)	$32\ x\ 32\ (1024)$
STN	$32\ x\ 32\ (1024)$
GPe	$32\ x\ 32\ (1024)$
CTX	$32\ x\ 32\ (1024)$

S2: Parameter Values of Neuronal Types

Table S2: Parameter values of the neuronal types used in the proposed model of LIT (Izhikevich, 2003; Humphries et al., 2009; Muddapu et al., 2019).

**Parameter(s)**	**STN**	**GPe**	**CTX**	**MSN**
Izhikevich parameters<br>$a\ (ms^{-1})$,<br>$b\ (pA.mV^{-1})$,	 $a = 0.005$,<br>$b = 0.265$,	 $a = 0.1$,<br>$b = 0.2$,	 $a = 0.03$,<br>$b = -2$,	 $a = 0.01$,<br>$b = -20$,

$c\ (mV),$<br>$d\ (pA)$	$c = -65,$<br>$d = 1.5$	$c = -65,$<br>$d = 2$	$c = -50,$<br>$d = 100$	$c = -55,$<br>$d = 91$
External current (I^x)	$3\ pA$	$4.25\ pA$	$100\ pA$	$0\ pA$
Maximum peak of voltage (v_{peak}^x)	$30\ mV$	$30\ mV$	$40\ mV$	$35\ mV$
Membrane capacitance (C^x)	$1\ \mu F$	$1\ \mu F$	$100\ \mu F$	$15.2\ pF$
Resting potential (v_r^x)	-	-	-	$-80\ mV$
Threshold potential (v_t^x)	-	-	-	$-29.7\ mV$
Membrane constant (k^x)	-	-	-	$1\ pA.mV^{-1}$
Number of laterals ($nlat^x$)	11	15	-	-
Radius of Gaussian laterals (R^x)	1.4	1.6	-	-
Synaptic strength within laterals (A^x)	1.3	0.1	-	-
Time decay constant for AMPA (τ_{AMPA})	$6\ ms$	$6\ ms$	$6\ ms$	$6\ ms$
Time decay constant for NMDA (τ_{NMDA})	$160\ ms$	$160\ ms$	$160\ ms$	$160\ ms$
Time decay constant for GABA (τ_{GABA})	$4\ ms$	$4\ ms$	$4\ ms$	$4\ ms$
Synaptic potential of AMPA receptor (E_{AMPA})	$0\ mV$	$0\ mV$	$0\ mV$	$0\ mV$
Synaptic potential of NMDA receptor (E_{NMDA})	$0\ mV$	$0\ mV$	$0\ mV$	$0\ mV$
Synaptic potential of GABA receptor (E_{GABA})	$-60\ mV$	$-60\ mV$	$-60\ mV$	$-60\ mV$
Concentration of Magnesium (Mg^{2+})	$1\ mM$	$1\ mM$	$1\ mM$	$1\ mM$

S3: SNc soma model (Muddapu and Chakravarthy, 2021)

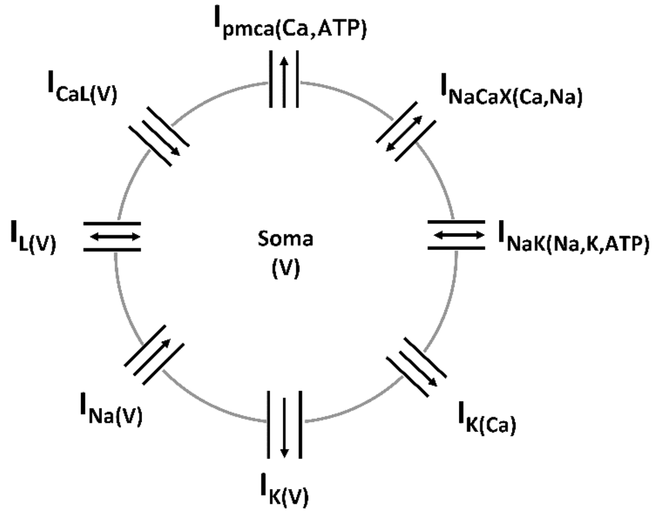

Figure S3: *Schematic of the single-compartment DA neuron model demonstrating the various ion currents in the model.*

The membrane potential equation for the SNc soma (V) is given by,

$$\frac{d(V)}{dt} = \frac{F * vol_{cyt}}{C_{snc} * AR_{pmu}} * \left[J_{m,Na} + 2 * J_{m,Ca} + J_{m,K} + J_{inp}\right] \tag{1}$$

where, F is the Faraday's constant, C_{snc} is the SNc membrane capacitance, vol_{cyt} is the cytosolic volume, AR_{pmu} is the cytosolic area, $J_{m,Na}$ is the sodium membrane ion flux, $J_{m,Ca}$ is the calcium membrane ion flux, $J_{m,K}$ is the potassium membrane ion flux, and J_{inp} is the overall input current flux.

Plasma Membrane Ion Channels

The intracellular calcium concentration dynamics ($[Ca_i]$) is given by,

$$\frac{d([Ca_i])}{dt} = J_{m,Ca} \tag{2}$$

$$J_{m,Ca} = -\frac{1}{z_{Ca} * F * vol_{cyt}} * \left(I_{CaL} + 2 * I_{pmca} - 2 * I_{NaCaX}\right) \tag{3}$$

where, z_{Ca} is the valence of calcium ion, I_{CaL} is the L-type calcium channel current, I_{pmca} is the ATP-dependent calcium pump current, I_{NaCaX} is the sodium-potassium exchanger current, F is the Faraday's constant, and vol_{cyt} is the cytosolic volume.

The voltage-dependent L-type calcium channel current (I_{CaL}) is given by,

$$I_{CaL}(V) = \left(\bar{g}_{Ca,L} * O_{Ca,L}\right) * \left(\sqrt{[Ca_i] * [Ca_e]}\right) * \left(\frac{\sinh(V_D - V_{Ca})}{\left(\frac{\sinh(V_D)}{V_D}\right)}\right) \tag{4}$$

$$O_{Ca,L} = m_{Ca,L} * h_{Ca,L} \tag{5}$$

where, $\bar{g}_{Ca,L}$ is the maximal conductance for calcium channel, $O_{Ca,L}$ is the gating variable of calcium channel, $m_{Ca,L}$ is the activation gate of the L-type calcium channel, $h_{Ca,L}$ is the inactivation gate of L-type calcium channel, $[Ca_i]$ is the intracellular calcium concentration, $[Ca_e]$ is the extracellular calcium concentration, V_{Ca} is the reversal potential for calcium ion, and V_D is the voltage defined thermodynamic entity.

$$\frac{d\left(m_{Ca,L}\right)}{dt} = \frac{\frac{1}{\left(1 + e^{\left(-\frac{(V+15)}{7}\right)}\right)} - m_{Ca,L}}{7.68 * e^{\left(-\left[\frac{V+65}{17.33}\right]^2\right)} + 0.723} \tag{6}$$

$$h_{Ca,L} = \frac{0.00045}{0.00045 + [Ca_i]} \tag{7}$$

The intracellular sodium concentration ($[Na_i]$) dynamics is given by,

$$\frac{d([Na_i])}{dt} = J_{m,Na} \tag{8}$$

$$J_{m,Na} = -\frac{1}{z_{Na} * F * vol_{cyt}} * (I_{NaT} + 3 * I_{NaK} + 3 * I_{NaCaX}) \quad (9)$$

where, z_{Na} is the valence of sodium ion, I_{NaT} is the total sodium channel current, I_{NaK} is the ATP-dependent sodium-potassium pump current, I_{NaCaX} is the sodium-potassium exchanger current, F is the Faraday's constant, and vol_{cyt} is the cytosolic volume.

The total sodium channel current is given by,

$$I_{NaT} = I_{Na} + I_{NaHCN} + I_{Nalk} \quad (10)$$

where, I_{Na} is the voltage-dependent sodium channel current, I_{NaHCN} is the hyperpolarization-activated cyclic nucleotide-gated sodium channel current, and I_{Nalk} is the leaky sodium channel current.

The voltage-dependent sodium channel current (I_{Na}) is given by,

$$I_{Na}(V) = (\bar{g}_{Na} * O_{Na}) * \left(\sqrt{[Na_i] * [Na_e]}\right) * \left(\frac{\sinh\left(\frac{1}{2} * (V_D - V_{Na})\right)}{\left(\frac{\sinh\left(\frac{1}{2} * V_D\right)}{\left(\frac{1}{2} * V_D\right)}\right)} \right) \quad (11)$$

$$O_{Na} = m_{Na}^3 * h_{Na} \quad (12)$$

where, $\bar{g}_{Na}$ is the maximal conductance for sodium channel, O_{Na} is the gating variable of sodium channel, m_{Na} is the activation gate of the sodium channel, h_{Na} is the inactivation gate of the sodium channel, $[Na_i]$ is the intracellular sodium concentration, $[Na_e]$ is the extracellular sodium concentration, V_{Na} is the reversal potential for sodium ion, and V_D is the voltage-defined thermodynamic entity.

$$\frac{d(m_{Na})}{dt} = 1.965 * e^{(1.7127*V_D)} * (1 - m_{Na}) - 0.0424 * e^{(-1.5581*V_D)} * (m_{Na}) \quad (13)$$

$$\frac{d(h_{Na})}{dt} = 0.00009566 * e^{(-2.4317*V_D)} * (1 - h_{Na}) - 0.5296 * e^{(1.1868*V_D)} * (h_{Na}) \quad (14)$$

The hyperpolarization-activated cyclic nucleotide (HCN) gated sodium channel current (I_{NaHCN}) is given by,

$$I_{NaHCN}(V) = (\bar{g}_{NaHCN} * O_{NaHCN}) * \left(\sqrt{[Na_i] * [Na_e]}\right) * \left(\frac{\sinh\left(\frac{1}{2} * (V_D - V_{Na})\right)}{\left(\frac{\sinh\left(\frac{1}{2} * V_D\right)}{\left(\frac{1}{2} * V_D\right)}\right)}\right) \quad (15)$$

where, $\bar{g}_{NaHCN}$ is the maximal conductance for sodium HCN channel, O_{NaHCN} is the gating variable of sodium HCN channel, $[Na_i]$ is the intracellular sodium concentration, $[Na_e]$ is the extracellular sodium concentration, V_{Na} is the reversal potential for sodium ion, V_D is the voltage defined thermodynamic entity, and $[cAMP]$ is the cyclic adenosine monophosphate concentration.

$$\frac{d(O_{NaHCN})}{dt} = k_{f,HCN} * (1 - O_{NaHCN}) - k_{r,HCN} * O_{NaHCN} \quad (16)$$

$$k_{f,HCN} = k_{f,free} * P_c + k_{f,bnd} * (1 - P_c) \quad (17)$$

$$k_{r,HCN} = k_{r,free} * P_o + k_{r,bnd} * (1 - P_o) \quad (18)$$

$$P_c = \frac{1}{\left(1 + \frac{[cAMP]}{0.001163}\right)}; \qquad P_o = \frac{1}{\left(1 + \frac{[cAMP]}{0.0000145}\right)} \quad (19)$$

$$k_{f,free} = \frac{0.006}{1 + e^{\left(\frac{V+87.7}{6.45}\right)}}; \qquad k_{f,bnd} = \frac{0.0268}{1 + e^{\left(\frac{V+94.2}{13.3}\right)}} \tag{20}$$

$$k_{r,free} = \frac{0.08}{1 + e^{\left(-\frac{V+51.7}{7}\right)}}; \qquad k_{r,bnd} = \frac{0.08}{1 + e^{\left(-\frac{V+35.5}{7}\right)}} \tag{21}$$

The leaky sodium channel current (I_{Nalk}) is given by,

$$I_{Nalk}(V) = (\bar{g}_{Nalk}) * \left(\sqrt{[Na_i] * [Na_e]}\right) * \left(\frac{\sinh\left(\frac{1}{2} * (V_D - V_{Na})\right)}{\left(\frac{\sinh\left(\frac{1}{2} * V_D\right)}{\left(\frac{1}{2} * V_D\right)}\right)}\right) \tag{22}$$

where, $\bar{g}_{Nalk}$ is the maximal conductance for leaky sodium channel, $[Na_i]$ is the intracellular sodium concentration, $[Na_e]$ is the extracellular sodium concentration, V_{Na} is the reversal potential for sodium ion, and V_D is the voltage defined thermodynamic entity.

The intracellular potassium concentration dynamics ($[K_i]$) is given by,

$$\frac{d([K_i])}{dt} = J_{m,K} \tag{23}$$

$$J_{m,K} = -\frac{1}{z_K * F * vol_{cyt}} * (I_{KT} - 2 * I_{NaK}) \tag{24}$$

where, z_K is the valence of potassium ion, I_{KT} is the total potassium channel current, I_{NaK} is the ATP-dependent sodium-potassium pump current, F is the Faraday's constant, and vol_{cyt} is the cytosolic volume.

The total potassium channel current is given by,

$$I_{KT} = I_{Kdr} + I_{Kir} + I_{Ksk} \tag{25}$$

where, I_{Kdr} is the voltage-dependent (delayed rectifying, DR) potassium channel current, I_{Kir} is the voltage-dependent (inward rectifying, IR) potassium channel current, and I_{Ksk} is the calcium-dependent (small conductance, SK) potassium channel current.

The voltage-dependent (delayed rectifying) potassium channel current (I_{Kdr}) is given by,

$$I_{Kdr}(V) = (\bar{g}_{Kdr} * O_{Kdr}) * (V - V_K * V_\tau) \quad (26)$$

$$O_{Kdr} = m_{Kdr}^3 \quad (27)$$

where, $\bar{g}_{Kdr}$ is the maximal conductance for delayed rectifying potassium channel, O_{Kdr} is the gating variable of voltage-dependent (delayed rectifying) potassium channel, V_K is the reversal potential for potassium ion, and V_τ is the temperature defined thermodynamic entity.

$$\frac{d(m_{K,dr})}{dt} = \frac{\frac{1}{\left(1 + e^{\left(-\frac{(V+25)}{12}\right)}\right)} - m_{K,dr}}{\frac{18}{\left(1 + e^{\left(-\left[\frac{V+65}{17.33}\right]^2\right)}\right)} + 1} \quad (28)$$

The voltage-dependent (inward rectifying) potassium channel current (I_{Kir}) is given by,

$$I_{Kir}(V) = (\bar{g}_{Kir} * O_{Kir}) * (V - V_K * V_\tau) \quad (29)$$

$$O_{Kir} = \frac{1}{\left(1 + e^{\left(\frac{V+85}{12}\right)}\right)} \quad (30)$$

where, $\bar{g}_{Kir}$ is the maximal conductance for inward rectifying potassium channel, O_{Kir} is the gating variable of voltage-dependent (inward rectifying) potassium channel, V_K is the reversal potential for potassium ion, and V_τ is the temperature defined thermodynamic entity.

The calcium-dependent (small conductance) potassium channel current (I_{Ksk}) is given by,

$$I_{Ksk}(V) = (\bar{g}_{Ksk} * O_{Ksk}) * \left(\sqrt{[K_i] * [K_e]}\right) * \left(\frac{\sinh\left(\frac{1}{2} * (V_D - V_K)\right)}{\left(\frac{\sinh\left(\frac{1}{2} * V_D\right)}{\left(\frac{1}{2} * V_D\right)}\right)} \right) \tag{31}$$

$$O_{Ksk} = \frac{[Ca_i]^{4.2}}{[Ca_i]^{4.2} + 0.00035^{4.2}} \tag{32}$$

where, $\bar{g}_{Ksk}$ is the maximal conductance for small conductance potassium channel, O_{Ksk} is the gating variable of calcium-dependent (small conductance) potassium channel, $[K_i]$ is the intracellular potassium concentration, $[K_e]$ is the extracellular potassium concentration, $[Ca_i]$ is the intracellular calcium concentration, V_K is the reversal potential for potassium ion, and V_D is the voltage defined thermodynamic entity.

The overall synaptic input current flux (J_{syn}) to SNc neuron is given by,

$$J_{syn} = -\frac{1}{F * vol_{cyt}} * \left(I_{syn}^{+} + I_{syn}^{-} - I_{ext}\right) \tag{33}$$

where, I_{syn}^{+} is the excitatory synaptic current, I_{syn}^{-} is the inhibitory synaptic current, I_{ext} is the external current applied, F is the Faraday's constant, and vol_{cyt} is the cytosolic volume. The different synaptic receptors were modeled similar to Destexhe et al.(Destexhe et al., 1998), and details are specified in *Supplementary Material-S7.*

Plasma Membrane ATPases

The plasma membrane sodium-potassium ATPase (I_{NaK}) is given by,

$$I_{NaK} = K_{nak} * \left[k_{1,nak} * \mathcal{P}\left(E_{1,nak}^{*}\right) * y_{nak} - k_{2,nak} * \mathcal{P}\left(E_{2,nak}^{*}\right) * (1 - y_{nak})\right] \tag{34}$$

$$\frac{d(y_{nak})}{dt} = \beta_{nak} * (1 - y_{nak}) - \alpha_{nak} * y_{nak} \quad (35)$$

$$\beta_{nak} = k_{2,nak} * \mathcal{P}\left(E_{2,nak}^{*}\right) + k_{4,nak} * \mathcal{P}\left(E_{2,nak}^{\#}\right) \quad (36)$$

$$\alpha_{nak} = k_{1,nak} * \mathcal{P}\left(E_{1,nak}^{*}\right) + k_{3,nak} * \mathcal{P}\left(E_{1,nak}^{\#}\right) \quad (37)$$

$$\mathcal{P}\left(E_{1,nak}^{*}\right) = \frac{1}{\left[1 + \frac{K_{nak,nai}}{[Na_i]} * \left(1 + \frac{[K_i]}{K_{nak,ki}}\right)\right]} \quad (38)$$

$$\mathcal{P}\left(E_{1,nak}^{\#}\right) = \frac{1}{\left[1 + \frac{K_{nak,ki}}{[K_i]} * \left(1 + \frac{[Na_i]}{K_{nak,nai}}\right)\right]} \quad (39)$$

$$\mathcal{P}\left(E_{2,nak}^{*}\right) = \frac{1}{\left[1 + \frac{K_{nak,nae}}{Na_{eff}} * \left(1 + \frac{[K_e]}{K_{nak,ke}}\right)\right]} \quad (40)$$

$$\mathcal{P}\left(E_{2,nak}^{\#}\right) = \frac{1}{\left[1 + \frac{K_{nak,ke}}{[K_e]} * \left(1 + \frac{Na_{eff}}{K_{nak,nae}}\right)\right]} \quad (41)$$

$$Na_{eff} = [Na_e] * e^{(-0.82 * V_D)} \quad (42)$$

$$k_{1,nak} = \frac{0.37}{1 + \frac{0.094}{[ATP_i]}} \quad (43)$$

where, K_{nak} is the maximal conductance for sodium-potassium ATPase, $[Na_i]$ is the intracellular concentration of sodium ion, $[Na_e]$ is the extracellular concentration of sodium ion, $[K_i]$ is the intracellular concentration of potassium ion, $[K_e]$ is the extracellular

concentration of potassium ion, $(k_{1,nak}, k_{2,nak}, k_{3,nak}, k_{4,nak})$ are the reaction rates, $(K_{nak,nae}, K_{nak,nai}, K_{nak,ke}, K_{nak,ki})$ are the dissociation constants, $[ATP_i]$ is the intracellular concentration of adenosine triphosphate (ATP), and V_D is the voltage defined thermodynamic entity.

The plasma membrane calcium ATPase (I_{pmca}) is given by,

$$I_{pmca} = K_{pc} * [k_{1,pc} * \mathcal{P}(E^*_{1,pc}) * y_{pc} - k_{2,pc} * \mathcal{P}(E^*_{2,pc}) * (1 - y_{pc})] \tag{44}$$

$$\frac{d(y_{pc})}{dt} = \beta_{pc} * (1 - y_{pc}) - \alpha_{pc} * y_{pc} \tag{45}$$

$$\beta_{pc} = k_{2,pc} * \mathcal{P}(E^*_{2,pc}) + k_{4,pc} * \mathcal{P}(E_{2,pc}) \tag{46}$$

$$\alpha_{pc} = k_{1,pc} * \mathcal{P}(E^*_{1,pc}) + k_{3,pc} * \mathcal{P}(E_{1,pc}) \tag{47}$$

$$\mathcal{P}(E^*_{1,pc}) = \frac{1}{\left(1 + \frac{K_{pc,i}}{[Ca_i]}\right)}; \qquad \mathcal{P}(E^*_{2,pc}) = \frac{1}{\left(1 + \frac{K_{pc,e}}{[Ca_e]}\right)} \tag{48}$$

$$\mathcal{P}(E_{1,pc}) = 1 - \mathcal{P}(E^*_{1,pc}); \qquad \mathcal{P}(E_{2,pc}) = 1 - \mathcal{P}(E^*_{2,pc}) \tag{49}$$

$$k_{1,pc} = \frac{1}{1 + \frac{0.1}{[ATP_i]}} \tag{50}$$

$$K_{pc,i} = \left[\frac{173.6}{1 + \frac{[CaCam]}{5 * 10^{-5}}} + 6.4\right] * 10^{-5} \tag{51}$$

$$K_{pc} = k_{pmca} * \left[\frac{10.56 * [CaCam]}{[CaCam] + 5 * 10^{-5}} + 1.2\right] \quad (52)$$

where, $(k_{1,pc}, k_{2,pc}, k_{3,pc}, k_{4,pc})$ are the reaction rates, k_{pmca} is the maximal conductance for calcium ATPase, $(K_{pc,e}, K_{pc,i})$ are the dissociation constants, $[ATP_i]$ is the intracellular concentration of ATP, $[Ca_i]$ is the intracellular calcium concentration, and $[CaCam]$ is the intracellular calcium-bound calmodulin concentration.

Plasma Membrane Exchangers

The plasma membrane sodium-calcium exchanger (I_{NaCaX}) is given by,

$$I_{NaCaX} = k_{xm} * \frac{[Na_i]^3 * [Ca_e] * exp^{(\delta_{xm} * V_D)} - [Na_e]^3 * [Ca_i] * e^{((\delta_{xm}-1) * V_D)}}{\left(1 + \mathcal{D}_{xm} * \left[[Na_i]^3 * [Ca_e] + [Na_e]^3 * [Ca_i]\right]\right) * \left(1 + \frac{[Ca_i]}{0.0069}\right)} \quad (53)$$

where, k_{xm} is the maximal conductance for sodium-calcium exchanger, $[Na_e]$ is the extracellular sodium concentration, $[Na_i]$ is the intracellular sodium concentration, $[Ca_e]$ is the extracellular calcium concentration, $[Ca_i]$ is the intracellular calcium concentration, δ_{xm} is the energy barrier parameter, $\mathcal{D}_{xm}$ is the denominator factor, and V_D is the voltage defined thermodynamic entity.

Table S3.1: Parameter values for SNc soma model (Francis et al., 2013).

Constant	Symbol	Value	Units
Faraday's constant	F	96485	$coulomb * mole^{-1}$
SNc membrane capacitance	C_{snc}	$9 \ x \ 10^7$	$pF * cm^{-2}$
Cytosolic volume	v_{cyt}	$\phi_{cyt} * v_{pmu}$	pl
Fraction of cytosolic volume	ϕ_{cyt}	0.5	$dimensionless$
Pacemaking unit (PMU) volume	v_{pmu}	5	pl
PMU area	$\mathcal{A}_{pmu}$	$\mathcal{S}_{pmu} * v_{pmu}$	cm^2

PMU surface area-to-volume ratio	S_{pmu}	$1.6667\ x\ 10^{4}$	cm^{-1}
Voltage defined thermodynamic entity	V_D	$\frac{V}{V_\tau}$	$dimensionless$
Temperature defined thermodynamic entity	V_τ	$\frac{R * T}{F}$	mV
Universal gas constant	R	8314.472	$mJ * mol^{-1} *\ K^{-1}$
Physiological temperature	T	310.15	K
Maximal conductance of calcium channel	$\bar{g}_{Ca,L}$	2101.2	$pA * mM^{-1}$
Extracellular calcium concentration	$[Ca_e]$	1.8	mM
Reversal potential for calcium ion	V_{Ca}	$\frac{1}{2} * \log\left(\frac{[Ca_e]}{[Ca_i]}\right)$	$dimensionless$
Valence of calcium ion	z_{Ca}	2	$dimensionless$
Maximal conductance of sodium channel	$\bar{g}_{Na}$	907.68	$pA * mM^{-1}$
Extracellular sodium concentration	$[Na_e]$	137	mM
Reversal potential for sodium ion	V_{Na}	$\log\left(\frac{[Na_e]}{[Na_i]}\right)$	$dimensionless$
Valence of sodium ion	z_{Na}	1	$dimensionless$
Maximal conductance of sodium HCN channel	$\bar{g}_{NaHCN}$	51.1	$pA * mM^{-1}$
Maximal conductance of leaky sodium channel	$\bar{g}_{Nalk}$	0.0053	$pA * mM^{-1}$
Cyclic adenosine monophosphate concentration	$[cAMP]$	$1\ x\ 10^{-5}$	mM

Maximal conductance of delayed rectifying potassium channel	$\bar{g}_{Kdr}$	31.237	nS
Extracellular potassium concentration	$[K_e]$	5.4	mM
Reversal potential for potassium ion	V_K	$\log\left(\frac{[K_e]}{[K_i]}\right)$	$dimensionless$
Valence of potassium ion	z_K	1	$dimensionless$
Maximal conductance of inward rectifying potassium channel	$\bar{g}_{Kir}$	13.816	nS
Maximal conductance of small conductance potassium channel	$\bar{g}_{Ksk}$	2.2515	$pA * mM^{-1}$
Maximal conductance for sodium-potassium ATPase	K_{nak}	1085.7	pA
Reaction rates of I_{NaK}	$k_{2,nak}$	0.04	ms^{-1}
	$k_{3,nak}$	0.01	ms^{-1}
	$k_{4,nak}$	0.165	ms^{-1}
Dissociation constants of I_{NaK}	$K_{nak,nae}$	69.8	mM
	$K_{nak,nai}$	4.05	mM
	$K_{nak,ke}$	0.258	mM
	$K_{nak,ki}$	32.88	mM
Maximal conductance for calcium ATPase	k_{pmca}	2.233	$pA * ms^{-1}$
Reaction rates of I_{pmca}	$k_{2,pc}$	0.001	ms^{-1}
	$k_{3,pc}$	0.001	ms^{-1}
	$k_{4,pc}$	1	ms^{-1}

Dissociation constants of I_{pmca}	$K_{pc,e}$	2	mM
Maximal conductance for sodium-calcium exchanger	k_{xm}	0.0166	$pA * ms^{-1}$
Energy barrier parameter of I_{NaCaX}	δ_{xm}	0.35	$dimensionless$
Denominator factor of I_{NaCaX}	$\mathcal{D}_{xm}$	0.001	$dimensionless$

Table S3.2: Steady state values of ion-channel dynamics of SNc cell model (Francis et al., 2013).

Symbol	Value	Symbol	Value
V	$-49.42\ mV$	h_{Na}	0.1848
$[Ca_i]$	$1.88\ x\ 10^{-4}\ mM$	O_{NaHCN}	0.003
$[Na_i]$	$4.69\ mM$	$m_{K,dr}$	0.003
$[K_i]$	$126.06\ mM$	y_{nak}	0.6213
m_{Na}	0.0952	y_{pc}	0.483

S4: SNc Terminal Model (Muddapu and Chakravarthy, 2021)

The three compartments are intracellular compartment representing cytosol, extracellular compartment representing extracellular space (ECS), and vesicular compartment representing a vesicle. In dopamine turnover processes, L-tyrosine (TYR) is converted into L-3,4-dihydroxyphenylalanine or L-DOPA by tyrosine hydroxylase (TH), which in turn is converted into dopamine (DA) by aromatic L-amino acid decarboxylase (AADC) *(Figure-S4.1)*. The cytoplasmic DA (DA_c) is stored into vesicles by vesicular monoamine transporter 2 (VMAT-2) *(Figure-S4.2)*. Upon arrival of action potential, vesicular DA (DA_v) is released into extracellular space *(Figure-S4.3)*. Most of the extracellular DA (DA_e) is taken up into the terminal through DA plasma membrane transporter (DAT) *(Figure-S4.4)* and remaining extracellular DA is metabolized by catechol-O-methyltransferase (COMT) and monoamine oxidase (MAO) into homovanillic acid (HVA) *(Figure-S4.5)*. The DA that enters the terminal

is again packed into vesicles, and the remaining cytoplasmic DA is metabolized by COMT and MAO enzymes *(Figure-S4.5)*. It is known that a DA neuron self-regulates its firing, neurotransmission and synthesis by autoreceptors (Anzalone et al., 2012; Ford, 2014). In the present model, we included autoreceptors that regulate the synthesis and release of dopamine *(Figure-S4.6, S4.7)*. Along with TYR, external L-DOPA compete for transporting into the terminal through aromatic L-amino acid transporter (AAT) *(Figure-S4.8)*.

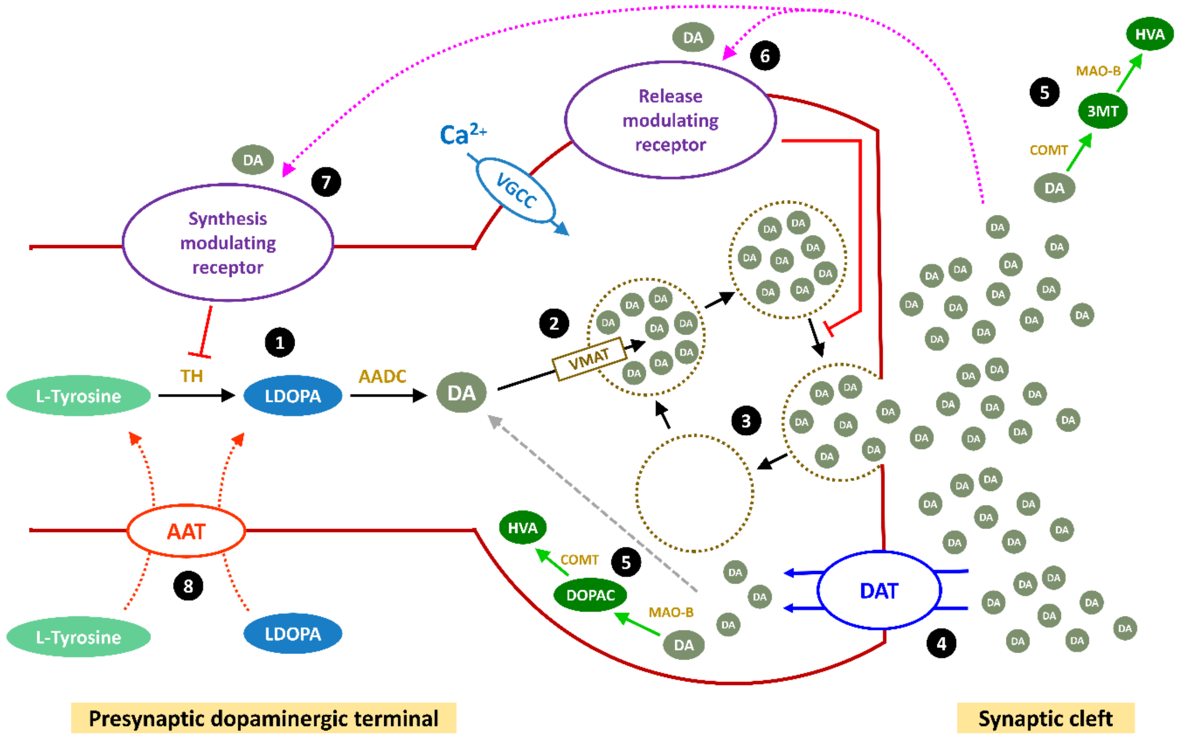

Figure S4: Schematic of Dopamine turnover processes in the SNc cell model.

<u>Modelling Extracellular DA in the ECS</u>

The major three mechanisms that determine the dynamics of extracellular DA ($[DA_e]$) in the ECS given by,

$$\frac{d([DA_e])}{dt} = J_{rel} - J_{DAT} - J_{eda}^{o} \quad (54)$$

where, J_{rel} represents the flux of calcium-dependent DA release from the DA terminal, J_{DAT} represents the unidirectional flux of DA translocated from the extracellular compartment (ECS) into the intracellular compartment (cytosol) via DA plasma membrane transporter (DAT), and J_{eda}^{o} represents the outward flux of DA degradation, which clears DA from ECS.

Calcium-Dependent DA Release Flux

Assuming that calcium-dependent DA release occurs within less than a millisecond after the calcium channels open, the flux of DA release (J_{rel}) from the DA terminal is given by,

$$J_{rel} = \psi * n_{RRP} * P_{rel}([Ca_i]) \tag{55}$$

where, $[Ca_i]$ is the intracellular calcium concentration in the DA terminal, P_{rel} is the release probability as a function of intracellular calcium concentration, n_{RRP} is the average number of readily releasable vesicles, and ψ is the average release flux per vesicle within a single synapse.

The flux of calcium-dependent DA release depends on extracellular DA concentration, and intracellular ATP acts as a feedback mechanism, assuming this regulation as extracellular DA and intracellular ATP controls the number of vesicles in the readily releasable vesicle pool (n_{RRP}) which is given by,

$$n_{RRP} = \frac{\eta_{nrrp} * e^{\left(\frac{[ATP_i]}{K_{a,RRP}}\right)}}{\left(1 + e^{\left[\frac{-([DA_v]-[DA_{v_o}])}{DA_{v_S}}\right]}\right) * \left(1 + e^{\left[\frac{[DA_e]-DA_{R_a}}{DA_{R_S}}\right]}\right)} \tag{56}$$

$$\eta_{nrrp} = \bar{\eta}_{nrrp} - \beta_{nrrp,asyn_{mis}} * \left(\frac{1}{1 + \left(\frac{K_{asyn_{mis}}}{[ASYN_{mis}]}\right)^4}\right) \tag{57}$$

where, $[DA_{v_o}]$ is the initial vesicular DA concentration, DA_{v_s} is the sensitivity to vesicular concentration, DA_{R_a} is the high-affinity state for DA binding to receptors and DA_{R_s} is the binding sensitivity, $[ATP_i]$ is the intracellular ATP concentration, $K_{a,RRP}$ is the activation constant for ATP, η_{nrrp} is the effect of misfolded alpha-synuclein on vesicle recycling (Venda et al., 2010), $\bar{\eta}_{nrrp}$ is the maximal vesicle recycling efficiency, $\beta_{nrrp,asyn_{mis}}$ is the maximum fractional decrease in the vesicle recycling efficiency through $ASYN_{mis}$, $K_{asyn_{mis}}$ is the threshold concentration for damage by $ASYN_{mis}$, and $[ASYN_{mis}]$ is the misfolded alpha-synuclein concentration.

The release probability of DA as a function of intracellular calcium concentration is given by,

$$P_{rel}([Ca_i]) = \bar{P}_{rel} * \frac{[Ca_i]^4}{[Ca_i]^4 + K_{rel}^4} \tag{58}$$

where, $\bar{P}_{rel}$ is the maximum release probability and K_{rel} is the sensitivity of calcium concentration, and $[Ca_i]$ is the intracellular calcium concentration.

Unidirectional Reuptake Flux of DA

The unidirectional reuptake flux of extracellular DA into the presynaptic terminal is given by,

$$J_{DAT} = \bar{V}_{eda} * \frac{[DA_e]}{K_{eda} + [DA_e]} \tag{59}$$

where, $\bar{V}_{eda}$ is the maximal velocity of dopamine transporter (DAT), K_{eda} is the DA concentration at half-maximal velocity, and $[DA_e]$ is the extracellular DA concentration.

Outward Extracellular Flux

The flux of extracellular DA enzymatic degradation in the synaptic cleft (ECS) is given by,

$$J_{eda}^{o} = k_{comt} * [DA_e] \tag{60}$$

where, k_{comt} is the rate at which extracellular DA cleared from ECS, and $[DA_e]$ is the extracellular DA concentration.

<u>*Modelling Intracellular DA in the Terminal*</u>

The intracellular DA dynamics ($[DA_i]$) is determined as the sum of dopamine concentration in cytosolic and vesicular compartments and is given by,

$$\frac{d([DA_i])}{dt} = \frac{d([DA_c])}{dt} + \frac{d([DA_v])}{dt} \tag{61}$$

The cytosolic DA dynamics ($[DA_c]$) is given by,

$$\frac{d([DA_c])}{dt} = J_{DAT} - J_{VMAT} - J_{cda}^{o} + J_{ldopa} \tag{62}$$

where, J_{DAT} represents the unidirectional flux of DA translocated from ECS into the cytosol through DAT, J_{VMAT} represents the flux of cytosolic DA into vesicle through VMAT-2, J_{ida}^{o} represents the outward flux of DA degradation, which clears DA from the cytosol, and J_{ldopa} represents the flux of synthesized cytosol DA from L-DOPA.

The vesicular DA dynamics ($[DA_v]$) is given by,

$$\frac{d([DA_v])}{dt} = J_{VMAT} - J_{rel} \tag{63}$$

where, J_{rel} represents the flux of calcium-dependent DA release from the DA terminal, J_{VMAT} represents the flux of cytosolic DA into a vesicle.

L-DOPA Synthesis Flux

The flux of synthesized L-DOPA whose velocity is the function of intracellular calcium concentration and L-DOPA synthesis is regulated by the substrate (TYR) itself, extracellular DA (via autoreceptor) and intracellular DA concentrations are given by,

$$J_{synt} = \frac{V_{synt}}{1 + \frac{K_{TYR}}{[TYR]} * \left(1 + \frac{[DA_c]}{K_{i,cda}} + \frac{[DA_e]}{K_{i,eda}}\right)} \tag{64}$$

where, V_{synt} is the velocity of synthesizing L-DOPA, $[TYR]$ is the tyrosine concentration in terminal bouton, K_{TYR} is the tyrosine concentration at which half-maximal velocity was attained, $K_{i,cda}$ is the inhibition constant on K_{TYR} due to cytosolic DA concentration, $K_{i,eda}$ is the inhibition constant on K_{TYR} due to extracellular DA concentration, $[DA_c]$ is the cytoplasmic DA concentration, and $[DA_e]$ is the extracellular DA concentration.

In Chen et al.(Chen et al., 2003), neuronal stimulation was linked to DA synthesis through an indirect event, which starts with calcium influx into the terminal bouton. In this model, the velocity of L-DOPA synthesis as a function of calcium levels in the terminal bouton is expressed as,

$$V_{synt}(Ca_i) = \bar{V}_{synt} * \frac{[Ca_i]^4}{K_{synt}^4 + [Ca_i]^4} \quad (65)$$

where, K_{synt} is the calcium sensitivity, $\bar{V}_{synt}$ is the maximal velocity for L-DOPA synthesis, and $[Ca_i]$ is the intracellular calcium concentration.

Storage Flux of DA into the Vesicle

The flux of transporting DA in the cytosol into the vesicles, which depends on the intracellular ATP is given by,

$$J_{VMAT} = V_{cda,ATP} * \frac{[DA_c]}{K_{cda} + [DA_c]} \quad (66)$$

$$V_{cda,ATP} = \bar{V}_{cda} * \alpha_{vmat} * e^{(\beta_{vmat} * [ATP_i])} \quad (67)$$

where, K_{cda} is the cytosolic DA concentration at which half-maximal velocity was attained, $\bar{V}_{cda}$ is the maximal velocity with which DA was packed into vesicles, $[DA_c]$ is the cytosolic DA concentration, α_{vmat} is the scaling factor for VMAT-2, β_{vmat} is the scaling factor for ATP_i, and $[ATP_i]$ is the intracellular ATP concentration.

Outward Intracellular Flux

The flux of intracellular DA enzymatic degradation in synaptic bouton (cytosol) is given by,

$$J_{cda}^{o} = k_{mao} * [DA_c] \quad (68)$$

where, k_{mao} is the rate at which intracellular DA cleared from the cytosol, and $[DA_c]$ is the cytosolic DA concentration.

L-DOPA to DA Conversion Flux

The flux of L-DOPA conversion to DA by AADC(Reed et al., 2012) is given by,

$$J_{ldopa} = \bar{V}_{aadc} * \frac{[LDOPA]}{K_{aadc} + [LDOPA]} \quad (69)$$

where, K_{aadc} is the L-DOPA concentration at which half-maximal velocity was attained, $\bar{V}_{aadc}$ is the maximal velocity with which L-DOPA was converted to DA, $[LDOPA]$ is the L-DOPA concentration.

Transport Flux of Exogenous L-DOPA into the Terminal

The flux of exogenous L-DOPA transported into the terminal through AAT while competing with other aromatic amino acids(Reed et al., 2012) is given by,

$$J_{aat} = \bar{V}_{aat} * \frac{[LDOPA_e]}{\left(K_{ldopa_e} * \left(1 + \left(\frac{[TYR_e]}{K_{tyr_e}}\right) + \left(\frac{[TRP_e]}{K_{trp_e}}\right)\right) + [LDOPA_e]\right)} \quad (70)$$

where, K_{ldopa_e} is the extracellular L-DOPA concentration at which half-maximal velocity was attained, $\bar{V}_{aat}$ is the maximal velocity with which extracellular L-DOPA was transported into the cytosol, $[LDOPA_e]$ is the extracellular L-DOPA concentration, $[TYR_e]$ is the extracellular TYR concentration, $[TRP_e]$ is the extracellular tryptophan (TRP) concentration, K_{tyr_e} is the affinity constant for $[TYR_e]$, K_{trp_e} is the affinity constant for $[TRP_e]$.

When L-DOPA drug therapy is initiated,

$$[LDOPA_e] = [sLD] \quad (71)$$

When no L-DOPA drug therapy is initiated,

$$LDOPA_e = 0 \quad (72)$$

The L-DOPA concentration ($[LDOPA]$) dynamics inside the terminal is given by,

$$\frac{d([LDOPA])}{dt} = J_{aat} - J_{ldopa} + J_{synt} \quad (73)$$

where, J_{aat} represents the flux of exogenous L-DOPA transported into the cytosol, J_{ldopa} represents the conversion flux of exogenous L-DOPA into DA, J_{synt} represents the flux of synthesized LDOPA from tyrosine, and $[sLD]$ is the serum L-DOPA concentration.

Table S4.1: Parameter values for DA turnover processes of SNc cell model (Reed et al., 2012; Tello-Bravo, 2012).

Constant	Symbol	Value	Units
Average release flux per vesicle	ψ	17.4391793	$mM * ms^{-1}$
Initial vesicular DA concentration	DA_{v_o}	500	mM
Sensitivity to vesicular DA concentration	DA_{v_s}	0.01	mM
Affinity constant of DA binding to receptors	DA_{R_a}	$5\ x\ 10^{-5}$	mM
Binding sensitivity	DA_{R_s}	0.01	mM
Activation constant for ATP	$K_{a,RRP}$	1.4286	mM
Vesicle recycling maximal flux	$\bar{v}_{nrrp}$	$1\ x\ 10^{-3}$	$mM * ms^{-1}$
Maximal vesicle recycling efficiency	$\bar{\eta}_{nrrp}$	0.995	$dimensionless$
Maximal fraction of $asyn^*$ effect on the vesicle	$\beta_{nrrp,asyn_{mis}}$	0.08	$dimensionless$
Affinity constant for $asyn^*$	$K_{asyn_{mis}}$	$8.5\ x\ 10^{-3}$	mM
Reaction constant of DA_e clearance	k_{comt}	0.0083511	ms^{-1}
Tyrosine concentration	$[TYR]$	$126\ x\ 10^{-3}$	mM
Affinity constant for TYR	K_{TYR}	$46\ x\ 10^{-3}$	mM
Inhibition constant for DA_c	$K_{i,cda}$	$11\ x\ 10^{-2}$	mM
Inhibition constant for DA_e	$K_{i,eda}$	$46\ x\ 10^{-3}$	mM
Maximal velocity of DA synthesis	$\bar{V}_{synt}$	$25\ x\ 10^{-6}$	$mM * ms^{-1}$
Affinity constant for Ca_i	K_{synt}	$35\ x\ 10^{-4}$	mM
Maximal velocity of VMAT	$\bar{V}_{cda}$	$4.67\ x\ 10^{-6}$	ms^{-1}

Affinity constant for DA_c	K_{cda}	$238\ x\ 10^{-4}$	mM
Scaling factor for VMAT	α_{vmat}	$1\ x\ 10^{-3}$	$dimensionless$
Scaling factor for ATP_i	β_{vmat}	3	$dimensionless$
Reaction constant of DA_c clearance	k_{mao}	0.00016	ms^{-1}
Maximal velocity of AADC	$\bar{V}_{aadc}$	$9.73\ x\ 10^{-5}$	$mM * ms^{-1}$
Affinity constant for $LDOPA$	K_{aadc}	0.13	mM
Maximal velocity of AAT	$\bar{V}_{aat}$	$5.11\ x\ 10^{-7}$	$mM * ms^{-1}$
Affinity constant for $LDOPA_e$	K_{ldopa_e}	$3.2\ x\ 10^{-4}$	mM
Affinity constant for TYR_e	K_{tyr_e}	$6.4\ x\ 10^{-4}$	mM
Affinity constant for TRP_e	K_{trp_e}	$1.5\ x\ 10^{-4}$	mM
Serum concentration of TYR	$[TYR_e]$	$6.3\ x\ 10^{-4}$	mM
Serum concentration of TRP	$[TRP_e]$	$8.2\ x\ 10^{-4}$	mM
Serum concentration of LDOPA	$[sLD]$	$3.6\ x\ 10^{-3}$	mM

Table S4.2: Steady state values of DA turnover processes of SNc cell model (Reed et al., 2012; Tello-Bravo, 2012).

**Symbol**	**Value**	**Symbol**	**Value**
$[DA_e]$	$4\ x\ 10^{-6}\ mM$	$[DA_v]$	$500\ mM$
$[DA_c]$	$1\ x\ 10^{-4}\ mM$	$[LDOPA]$	$3.6\ x\ 10^{-4}\ mM$

S5: Connectivity in the Model

Table S5: Connectivity patterns in the proposed LIT model (Oorschot, 1996).

From – to	Pattern (signal)
SNc (soma) – SNc (terminal)	1 to 16 (Calcium)
SNc (terminal) – D1-MSN (GS)	20 to 1 (Dopamine)
SNc (terminal) – D1-MSN (G)	20 to 1 (Dopamine)
D1-MSN (GS) – D1-MSN (G)	1 to 1 (GABA & SP)
D1-MSN (G) – D1-MSN (GS)	1 to 1 (GABA)
D1-MSN (GS) – SNc (soma)	200 to 1 (GABA & SP)
D1-MSN (G) – SNc (soma)	200 to 1 (GABA)
STN – GPe	1 to 1 (Glutamate)
GPe – STN	1 to 1 (GABA)
STN – SNc (soma)	16 to 1 (Glutamate)
CTX – D1-MSN (GS)	1 to 1 (Glutamate)
CTX – D1-MSN (G)	1 to 1 (Glutamate)
STN – STN	Gaussian neighborhoods (Glutamate)
GPe – GPe	Gaussian neighborhoods (GABA)
SNc – SNc	Gaussian neighborhoods (GABA)

S6: Parameter Values of Connectivity

Table S6: Parameter values of the connectivity used in the proposed model of LIT (Terman et al., 2002; Reed et al., 2012; Buxton et al., 2017).

Parameter	Value	Parameter	Value
Number of laterals ($nlat^x$)	5	θ_g	$20\ mV$
Radius of Gaussian laterals (R^x)	1.6	θ_g^H	$-57\ mV$

Synaptic strength within laterals (A^x)	0.1	σ_g^H	$2\ mV$
Synaptic conductance $\left(W_{x\to y}\right)$	0.01	α	$2\ ms^{-1}$
Synaptic potential of GABA receptor (E_{GABA})	$63.45\ mV$	β	$0.08\ ms^{-1}$
s_{max}^{STN}	1.3	cd_{stn}	4.87
s_{min}^{GPe}	0.1	cd_{gpe}	7
s_{min}^{SNc}	$1\ x10^{-6}$	cd_{snc}	4.6055
$cd2$	0.1	w_{sp}	5000
K^{MSN}	0.0289	L^{MSN}	0.331
$\alpha_{DA}^{D1-MSN(G)}$	1	$\alpha_{DA}^{D1-MSN(GS)}$	2
$w_{GPe\to GPe}$	1	$w_{SNc\to SNc}$	0.01
$w_{STN\to GPe}$	1	$w_{GPe\to STN}$	20
$w_{STN\to STN}$	1.3	$w_{STN\to SNc}$	0.3
$w_{D1-MSN\ (G)\to SNc}$	0.5	$w_{D1-MSN\ (GS)\to SNc}$	0.5
$w_{D1-MSN\ (G)\to D1-MSN\ (GS)}$	500	$w_{CTX\to D1-MSN\ (GS)}$	100
$w_{CTX\to D1-MSN\ (G)}$	100	τ_d^{sp}	$40\ ms$
τ_f^{sp}	$200\ ms$	τ_r^{sp}	$10\ ms$
β_{sp}	0.47	λ_{sp}	5.5
b_{sp}	2.5	$F_{STN\to SNc}$	$1\ x10^{-5}$
$F_{D1-MSN\ (G)\to SNc}$	$4.15\ x10^{-6}$	$F_{D1-MSN\ (GS)\to SNc}$	$4.15\ x10^{-6}$
V_{trans}^{max}	$5.11\ x10^{-7}\ mM.ms^{-1}$	$K_m^{LDOPA_S}$	$0.032\ mM$
$[TYR_s]$	$0.063\ mM$	$K_a^{TYR_s}$	$0.064\ mM$

$[TRP_s]$	$0.082\ mM$	$K_a^{TRP_s}$	$0.015\ mM$
P_{soma}^{snc}	64	$P_{terminal}^{snc}$	1024
ER_{thres}	$2.15\ x10^{-3}\ mM$	ROS_{thres}	$0.0147\ mM$
MT_{thres}	$0.0215\ mM$		

S7: Receptor model (Destexhe et al., 1998)

AMPA/Kainate Receptors

The simplest model that approximates the kinetics of the fast AMPA/kainate type of glutamate receptors can be represented by the two-state diagram:

$$C + T \overset{(\alpha/\beta)}{\Longleftrightarrow} O \tag{74}$$

where, α and β are voltage-independent forward and backward rate constants, C is the closed state of the receptor, O is the open state of the receptor, and T is the neurotransmitter. If r is defined as the fraction of the receptors in the open state, it is then described by the following first-order kinetic equation:

$$\frac{d(r)}{dt} = \alpha * [T] * (1 - r) - \beta * r \tag{75}$$

and the postsynaptic current (I_{AMPA}) is given by,

$$I_{AMPA} = \bar{g}_{AMPA} * r * (V - E_{AMPA}) \tag{76}$$

where, $\bar{g}_{AMPA}$ is the maximal conductance, E_{AMPA} is the reversal potential, V is the postsynaptic membrane potential, $[T]$ is the neurotransmitter, and r is the fraction of the receptors in the open state.

NMDA Receptors

The slower NMDA type of glutamate receptors can be represented with a two-state model similar to AMPA/kainate receptors, with a voltage-dependent term representing magnesium block. Using the scheme in Eqs. 1 and 2, the postsynaptic current is given by

$$I_{NMDA} = \bar{g}_{NMDA} * r * B(V) * (V - E_{NMDA}) \tag{77}$$

where, $\bar{g}_{NMDA}$ is the maximal conductance, E_{NMDA} is the reversal potential, $B(V)$ is the magnesium block, V is the postsynaptic membrane potential, and r is the fraction of the receptors in the open state.

$$B(V) = \frac{1}{1 + \left(\frac{[Mg^{2+}]}{3.57} * e^{-0.062 * V}\right)} \tag{78}$$

where, $[Mg^{2+}]$ is the external magnesium concentration, and V is the postsynaptic membrane potential.

GABA$_A$ Receptors

GABA$_A$ receptors can also be represented by the scheme in Eqs. 1 and 2, with the postsynaptic current given by

$$I_{GABA_A} = \bar{g}_{GABA_A} * r * \left(V - E_{GABA_A}\right) \tag{79}$$

where, $\bar{g}_{GABA_A}$ is the maximal conductance, E_{GABA_A} is the reversal potential, V is the postsynaptic membrane potential, and r is the fraction of the receptors in the open state.

GABA$_B$ Receptors

The stimulus dependency of GABA$_B$ responses, unfortunately, cannot be handled correctly by a two-state model. The simplest model of GABA$_B$-mediated currents has two variables:

$$\frac{d(r)}{dt} = K_1 * [T] * (1 - r) - K_2 * r \tag{80}$$

$$\frac{d(s)}{dt} = K_3 * r - K_4 * s \tag{81}$$

and the postsynaptic current $\left(I_{GABA_B}\right)$ is given by,

$$I_{GABA_B} = \bar{g}_{GABA_B} * \frac{s^n}{s^n + K_d} * \left(V - E_{GABA_B}\right) \quad (82)$$

where, $\bar{g}_{GABA_B}$ is the maximal conductance, E_{GABA_B} $(= V_K)$ is the reversal potential, V is the postsynaptic membrane potential, r is the fraction of the receptors in the open state, s is the fraction of activated G-proteins, K_d is the dissociation constant of the binding of s on the K^+ channels, K_1 and K_2 are voltage-independent forward and backward rate constants for r, K_3 and K_4 are voltage-independent forward and backward rate constants for s, and $[T]$ is the neurotransmitter.

Overall Synaptic Current

The overall synaptic input current flux $\left(J_{syn}\right)$ to SNc neuron is given by,

$$J_{syn} = -\frac{1}{F * v_{cyt}} * \left(I_{AMPA} + I_{NMDA} + I_{GABA_A} + I_{GABA_B}\right) \quad (83)$$

where, I_{AMPA} is the excitatory AMPA synaptic current, I_{NMDA} is the excitatory NMDA synaptic current, I_{GABA_A} is the inhibitory $GABA_A$ synaptic current, I_{GABA_B} is the inhibitory $GABA_B$ synaptic current, F is the Faraday's constant, and v_{cyt} is the cytosolic volume.

Table S7: Parameter values of receptor models (Destexhe et al., 1998; Francis et al., 2013)

Constant	Symbol	Value	Units
Faraday's constant	F	96485	$coulomb * mole^{-1}$
Cytosolic volume	v_{cyt}	$\phi_{cyt} * v_{pmu}$	pl
Fraction of cytosolic volume	ϕ_{cyt}	0.5	$dimensionless$
Pacemaking unit (PMU) volume	v_{pmu}	5	pl

Maximal conductance of AMPA receptor	$\bar{g}_{AMPA}$	$0.35-1$	nS
Maximal conductance of NMDA receptor	$\bar{g}_{NMDA}$	$0.01-0.6$	nS
Concentration of Magnesium	$[Mg^{2+}]$	$1-2$	mM
Maximal conductance of $GABA_A$ receptor	$\bar{g}_{GABA_A}$	$0.25-1.2$	nS
Maximal conductance of $GABA_B$ receptor	$\bar{g}_{GABA_B}$	0.06	nS
Dissociation constant of the binding of s on the K^+ channels	K_d	100	μM^4
Voltage-independent forward rate constant for r of $GABA_B$	K_1	$9\ x\ 10^4$	$M^{-1} * sec^{-1}$
Voltage-independent backward rate constant for r of $GABA_B$	K_2	1.2	sec^{-1}
Voltage-independent forward rate constant for s of $GABA_B$	K_3	180	sec^{-1}
Voltage-independent backward rate constant for s of $GABA_B$	K_4	34	sec^{-1}
Cooperativity constant (binding sites)	n	4	$dimensionless$
Reversal potential of AMPA	E_{AMPA}	0	mV
Reversal potential of NMDA	E_{NMDA}	0	mV
Reversal potential of $GABA_A$	E_{GABA_A}	-80	mV
Reversal potential of $GABA_B$	E_{GABA_B}	-95	mV
	AMPA	$1.1\ x\ 10^6$	$M^{-1} * sec^{-1}$

Voltage-independent forward rate constant for r (α)	NMDA	$7.2 \; x \; 10^4$	$M^{-1} * sec^{-1}$
	GABA$_A$	$5 \; x \; 10^6$	$M^{-1} * sec^{-1}$
Voltage-independent backward rate constant for r (β)	AMPA	190	sec^{-1}
	NMDA	6.6	sec^{-1}
	GABA$_A$	180	sec^{-1}

S8: Calcium Dynamics (Muddapu and Chakravarthy, 2021)

Intracellular calcium plays an essential role in the normal functioning of the cell. In order to maintain calcium homeostasis, the intracellular calcium levels are tightly regulated by calcium buffering mechanisms such as calcium-binding proteins, endoplasmic reticulum (ER), and mitochondria (MT) (Alzheimer, 2003).

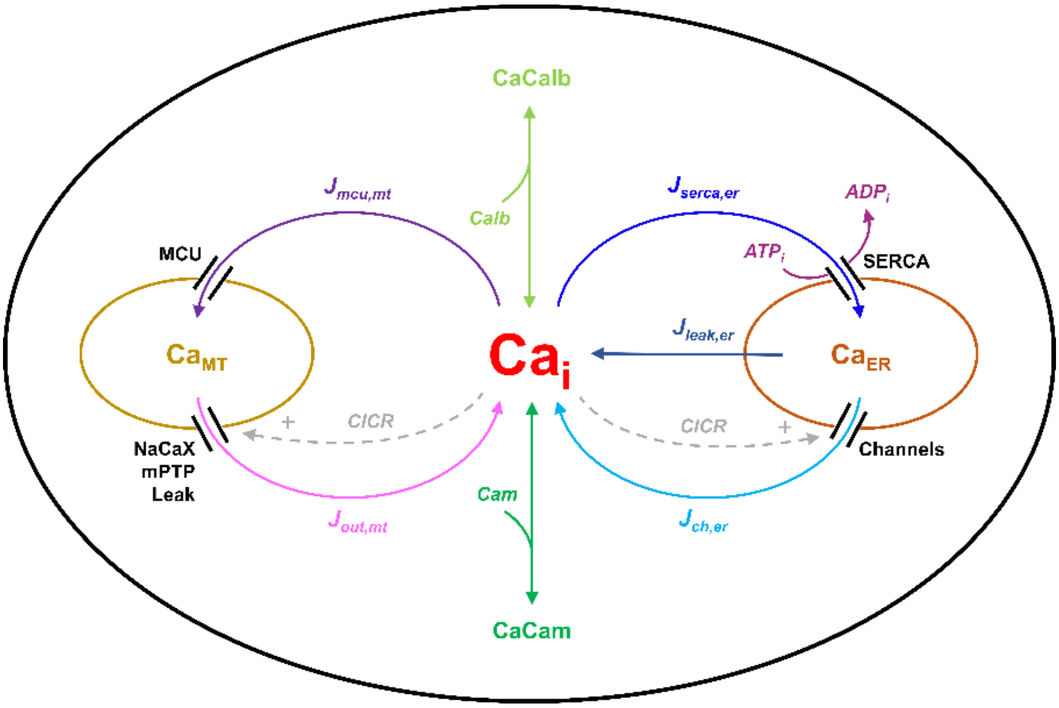

Figure S8: Schematic of calcium dynamics in the SNc cell model.

The intracellular calcium concentration dynamics ($[Ca_i]$) after including calcium buffering mechanisms (Marhl et al., 2000; Francis et al., 2013) (Figure S8) is given by,

$$\frac{d([Ca_i])}{dt} = J_{m,Ca} - J_{calb} - 4 * J_{cam} - J_{serca,er} + J_{ch,er} + J_{leak,er} - J_{mcu,mt} + J_{out,mt} \tag{84}$$

where, $J_{m,Ca}$ is the flux of calcium ion channels, J_{calb} is the calcium buffering flux by calbindin, J_{cam} is the calcium buffering flux by calmodulin, $J_{serca,er}$ is the calcium buffering flux by ER uptake of calcium through sarco/endoplasmic reticulum calcium-ATPase (SERCA), $J_{ch,er}$ is the calcium efflux from ER by calcium-induced calcium release (CICR) mechanism, $J_{leak,er}$ is the calcium leak flux from ER, $J_{mcu,mt}$ is the calcium buffering flux by MT uptake of calcium through mitochondrial calcium uniporters (MCUs), and $J_{out,mt}$ is the calcium efflux from MT through sodium-calcium exchangers, mitochondrial permeability transition pores (mPTPs), and non-specific leak flux.

The calcium buffering flux by calbindin (J_{calb}) is given by,

$$J_{calb} = k_{1,calb} * [Ca_i] * [Calb] - k_{2,calb} * [CaCalb] \tag{85}$$

$$[CaCalb] = [Calb_{tot}] - [Calb] \tag{86}$$

$$\frac{d([Calb])}{dt} = -J_{calb} \tag{87}$$

where, $(k_{1,calb}, k_{2,calb})$ are the calbindin reaction rates, $[Ca_i]$ is the intracellular calcium concentration, $[Calb]$ is the calbindin concentration, $[CaCalb]$ is the calcium-bound calbindin concentration, and $[Calb_{tot}]$ is the total cytosolic calbindin concentration.

The calcium buffering flux by calmodulin (J_{cam}) is given by,

$$J_{cam} = \alpha_{cam} * [Cam] - \beta_{cam} * [CaCam] \tag{88}$$

$$[CaCam] = [Cam_{tot}] - [Cam] \tag{89}$$

$$\frac{d([Cam])}{dt} = -J_{cam} \tag{90}$$

$$\alpha_{cam} = K_{cam}^{cb} * K_{cam}^{nb} * \left[\frac{1}{K_{cam}^{cb} + k_{cam}^{nd}} + \frac{1}{k_{cam}^{cd} + k_{cam}^{nd}}\right] \tag{91}$$

$$\beta_{cam} = k_{cam}^{cd} * k_{cam}^{nd} * \left[\frac{1}{K_{cam}^{cb} + k_{cam}^{nd}} + \frac{1}{k_{cam}^{cd} + k_{cam}^{nd}}\right] \tag{92}$$

$$K_{cam}^{cb} = k_{cam}^{cb} * [Ca_i]^2; \qquad K_{cam}^{nb} = k_{cam}^{nb} * [Ca_i]^2 \tag{93}$$

where, $(k_{cam}^{nd}, k_{cam}^{cd}, k_{cam}^{cb}, k_{cam}^{cb})$ are the calmodulin reaction rates, $[Ca_i]$ is the intracellular calcium concentration, $[Cam]$ is the calmodulin concentration, $[CaCam]$ is the calcium-bound calmodulin concentration, and $[Cam_{tot}]$ is the total cytosolic calmodulin concentration.

The calcium buffering flux by ER uptake of calcium through SERCA $(J_{serca,er})$ is given by,

$$J_{serca,er} = k_{serca,er} * [Ca_i] * [ATP_i] \tag{94}$$

where, $k_{serca,er}$ is the maximal rate constant of SERCA, $[Ca_i]$ is the intracellular calcium concentration, and $[ATP_i]$ is the intracellular ATP concentration.

The calcium efflux from ER by CICR $(J_{cicr,er})$ is given by,

$$J_{ch,er} = k_{cicr,er} * \left(\frac{[Ca_i]^2}{K_{cicr,er}^2 + [Ca_i]^2}\right) * ([Ca_{er}] - [Ca_i]) \tag{95}$$

where, $k_{ch,er}$ is the maximal permeability of calcium channels in the ER membrane, $K_{ch,er}$ is the half-saturation for calcium, $[Ca_i]$ is the intracellular calcium concentration, and $[Ca_{er}]$ is the ER calcium concentration.

The calcium leak flux from ER $(J_{leak,er})$ is given by,

$$J_{leak,er} = k_{leak,er} * ([Ca_{er}] - [Ca_i]) \tag{96}$$

where, $k_{leak,er}$ is the maximal rate constant for calcium leak flux through the ER membrane, $[Ca_i]$ is the intracellular calcium concentration, and $[Ca_{er}]$ is the ER calcium concentration.

The ER calcium concentration ($[Ca_{er}]$) dynamics is given by,

$$\frac{d([Ca_{er}])}{dt} = \frac{\beta_{er}}{\rho_{er}} * \left(J_{serca,er} - J_{ch,er} - J_{leak,er}\right) \tag{97}$$

where, β_{er} is the ratio of free calcium to total calcium concentration in the ER, ρ_{er} is the volume ratio between the ER and cytosol, $J_{serca,er}$ is the calcium buffering flux by ER uptake of calcium through SERCA, $J_{ch,er}$ is the calcium efflux from ER by CICR mechanism, and $J_{leak,er}$ is the calcium leak flux from ER.

The calcium buffering flux by MT uptake of calcium through MCUs $\left(J_{mcu,mt}\right)$ is given by,

$$J_{mcu,mt} = k_{mcu,mt} * \left(\frac{[Ca_i]^8}{K_{mcu,mt}^8 + [Ca_i]^8}\right) \tag{98}$$

where, $k_{mcu,mt}$ is the maximal permeability of mitochondrial membrane calcium uniporters, $K_{mcu,mt}$ is the half-saturation for calcium, and $[Ca_i]$ is the intracellular calcium concentration.

The calcium efflux from MT through sodium-calcium exchangers, mPTPs, and non-specific leak flux $\left(J_{out,mt}\right)$ is given by,

$$J_{out,mt} = \left(k_{out,mt} * \left(\frac{[Ca_i]^2}{K_{out,mt}^2 + [Ca_i]^2}\right) + k_{leak,mt}\right) * [Ca_{mt}] \tag{99}$$

where, $k_{out,mt}$ is the maximal rate of calcium flux through sodium-calcium exchangers and mitochondrial permeability transition pores, $K_{out,mt}$ is the half-saturation for calcium, $k_{leak,mt}$ is the maximal rate constant for calcium leak flux through the MT membrane, $[Ca_i]$ is the intracellular calcium concentration, and $[Ca_{mt}]$ is the MT calcium concentration.

The MT calcium concentration ($[Ca_{mt}]$) dynamics is given by,

$$\frac{d([Ca_{mt}])}{dt} = \frac{\beta_{mt}}{\rho_{mt}} * (J_{mcu,mt} - J_{out,mt}) \tag{100}$$

where, β_{mt} is the ratio of free calcium to total calcium concentration in the ER, ρ_{mt} is the volume ratio between the MT and cytosol, $J_{mcu,mt}$ is the calcium buffering flux by MT uptake of calcium through MCUs, and $J_{out,mt}$ is the calcium efflux from MT through sodium-calcium exchangers, mPTPs, and non-specific leak flux.

The total instantaneous concentration of calcium ($[Ca_{tot}]$) in the SNc cell at a given time t is given by,

$$[Ca_{tot}](t) = [Ca_i](t) + \frac{\rho_{er}}{\beta_{er}} * [Ca_{er}](t) + \frac{\rho_{mt}}{\beta_{mt}} * [Ca_{mt}](t) + [CaCalb](t) + [CaCam](t) \tag{101}$$

where, β_{er} is the ratio of free calcium to total calcium concentration in the ER, ρ_{er} is the volume ratio between the ER and cytosol, β_{mt} is the ratio of free calcium to total calcium concentration in the ER, ρ_{mt} is the volume ratio between the MT and cytosol, $[Ca_i](t)$, $[Ca_{er}](t)$, $[Ca_{mt}](t)$, $[CaCalb](t)$, and $[CaCam](t)$ are the instantaneous concentration of intracellular (cytoplasmic) calcium, ER calcium, MT calcium, calcium-bound calbindin, and calcium-bound calmodulin, respectively.

Table S8.1: Parameter values of calcium buffering mechanisms of SNc cell model (Marhl et al., 2000; Francis et al., 2013).

**Constant**	**Symbol**	**Value**	**Units**
Calbindin reaction rates	$k_{1,calb}$	10	$mM^{-1} * ms^{-1}$
	$k_{2,calb}$	$2\ x\ 10^{-3}$	ms^{-1}
Total cytosolic calbindin concentration	$[Calb_{tot}]$	0.005	mM
Calmodulin reaction rates	k_{cam}^{cb}	12000	$mM^{-2} * ms^{-1}$

	k_{cam}^{nb}	$3.7\ x\ 10^{6}$	$mM^{-2} * ms^{-1}$
	k_{cam}^{cd}	$3\ x\ 10^{-3}$	ms^{-1}
	k_{cam}^{nd}	3	ms^{-1}
Total cytosolic calmodulin concentration	$[Cam_{tot}]$	0.0235	mM
The maximal rate constant of SERCA	$k_{serca,er}$	0.02	$mM^{-1} * ms^{-1}$
Maximal permeability of calcium channels in the ER membrane	$k_{ch,er}$	3	ms^{-1}
Half saturation for calcium	$K_{ch,er}$	0.005	mM
Maximal rate constant for calcium leak flux through the ER membrane	$k_{leak,er}$	$5\ x\ 10^{-5}$	ms^{-1}
Ratio of free calcium to total calcium concentration in ER	β_{er}	0.0025	$dimensionless$
Volume ratio between the ER and cytosol	ρ_{er}	0.01	$dimensionless$
Maximal permeability of MCUs	$k_{mcu,mt}$	$3\ x\ 10^{-4}$	$mM * ms^{-1}$
Half saturation for calcium	$K_{mcu,mt}$	$8\ x\ 10^{-4}$	mM
Maximal rate of calcium flux through $[Na^{+}]/[Ca^{2+}]$ exchangers and mPTPs	$k_{out,mt}$	0.125	ms^{-1}
Half saturation for calcium	$K_{out,mt}$	0.005	mM
Maximal rate constant for calcium leak flux through the MT membrane	$k_{leak,mt}$	$6.25\ x\ 10^{-6}$	ms^{-1}

Ratio of free calcium to total calcium concentration in MT	β_{mt}	0.0025	$dimensionless$
Volume ratio between the MT and cytosol	ρ_{mt}	0.01	$dimensionless$

Table S8.2: Steady state values of calcium buffering mechanisms of SNc cell model (Marhl et al., 2000; Francis et al., 2013).

**Symbol**	**Value**	**Symbol**	**Value**
$[Ca_{er}]$	$1\ x\ 10^{-3}\ mM$	$[Calb]$	$26\ x\ 10^{-4}\ mM$
$[Ca_{mt}]$	$4\ x\ 10^{-4}\ mM$	$[Cam]$	$222\ x\ 10^{-4}\ mM$

S8: Molecular Pathways Involved in PD Pathology (Muddapu and Chakravarthy, 2021)

ROS formation occurs due to leakage from mitochondria during oxidative phosphorylation for ATP production, auto-oxidation of excess freely available DA in the cytoplasm, and misfolded alpha-synuclein ($ASYN_{mis}$). In the present model, excess ROS is scavenged by glutathione. Under pathological conditions such as elevated ROS levels, normal alpha-synuclein ($ASYN$) undergoes conformation changes into misfolded alpha-synuclein. The misfolded alpha-synuclein is tagged ($ASYN_{tag}$) and degraded by the ubiquitous-proteasome pathway using ATP. Excess misfolded alpha-synuclein forms aggregates, which in turn gets degraded by the lysosomal degradation pathway using ATP. In some scenarios, these alpha-synuclein aggregates ($ASYN_{agg}$) form Lewy bodies (LBs).

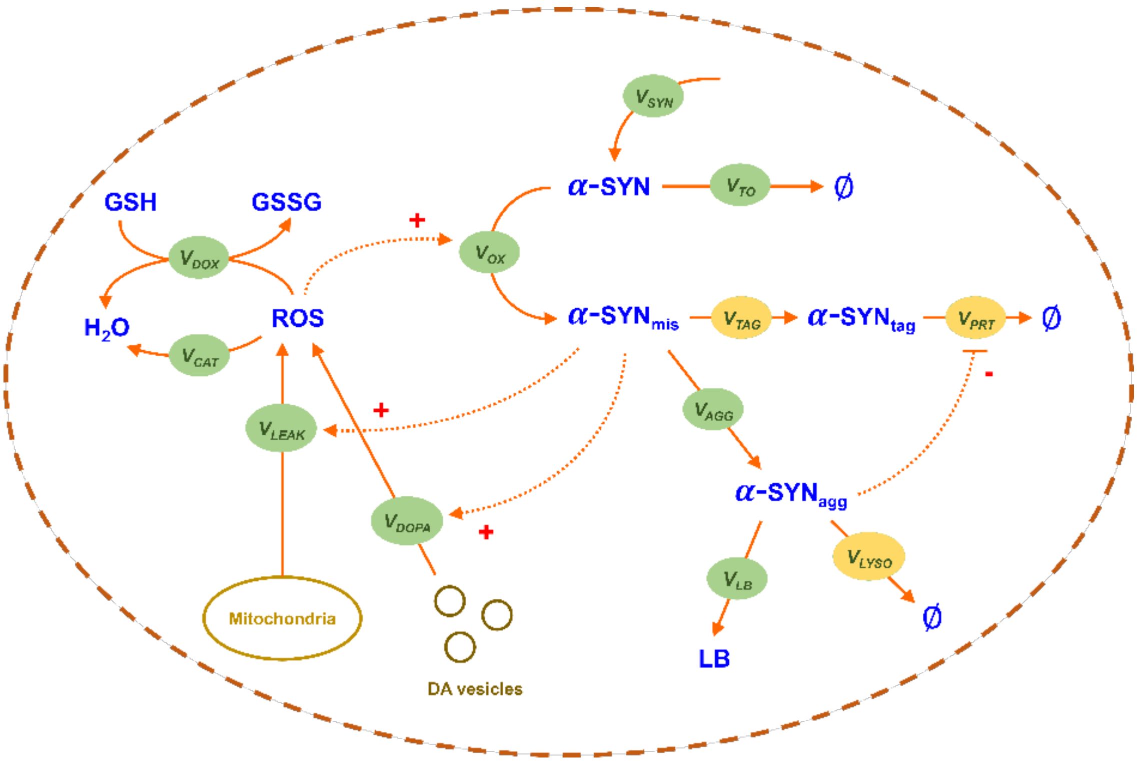

Figure S9: Schematic of molecular pathways in PD pathology in the SNc cell model.

The model consists of ROS formation from different processes, including ROS scavenging mechanism, alpha-synuclein aggregation, proteasomal and lysosomal degradation of damaged protein, etc. The following equations give a concise view of all metabolite dynamics in the PD pathology pathways,

Reactive oxygen species: $$\frac{d([ROS])}{dt} = J_{leak} + J_{env} + J_{dopa} - J_{cat} - J_{dox} \qquad (102)$$

Alpha-synuclein: $$\frac{d([ASYN])}{dt} = J_{syn} - J_{ox} - J_{to} \qquad (103)$$

Misfolded alpha-synuclein: $$\frac{d([ASYN_{mis}])}{dt} = J_{ox} - J_{agg} - J_{tag} \qquad (104)$$

Tagged alpha-synuclein: $$\frac{d([ASYN_{tag}])}{dt} = J_{tag} - J_{prt} \qquad (105)$$

Aggregated alpha-synuclein: $$\frac{d([ASYN_{agg}])}{dt} = J_{agg} - J_{lyso} - J_{lb} \quad (106)$$

Lewy bodies: $$\frac{d([LB])}{dt} = J_{lb} \quad (107)$$

where, J_{leak} is the flux of oxidative stress due to mitochondrial leakage, J_{env} is the flux of external oxidative stress (includes environmental toxins, inflammatory responses, etc.), J_{dopa} is the flux of oxidative stress due to excess cytoplasmic dopamine, J_{cat} is the catabolizing flux of ROS by catalase enzyme, J_{dox} is the flux of GSH-dependent ROS scavenging pathway (Eq. 104), J_{syn} is the synthesizing flux of alpha-synuclein protein, J_{ox} is the flux of alpha-synuclein misfolding due to ROS, J_{to} is the usage flux of alpha-synuclein in other processes, J_{agg} is the flux of alpha-synuclein aggregation, J_{tag} is the flux of ATP-dependent ubiquitination of damaged protein for proteasomal degradation, J_{prt} is the flux of ATP-dependent breakdown of damaged protein through proteasomal degradation, J_{lyso} is the flux of ATP-dependent breakdown of aggregated protein through lysosomal degradation, and J_{lb} is the flux of LBs formation.

The flux of oxidative stress due to mitochondrial leakage (J_{leak}) is given by,

$$J_{leak} = \left(\frac{K_{a,leak}}{[ATP_i]}\right) * \left(1 - \eta_{op}\right) * J_{op} \quad (108)$$

where, J_{op} is the flux of the oxidative phosphorylation pathway, η_{op} is the electron transport chain efficiency, $[ATP_i]$ is the intracellular ATP concentration, and $K_{a,ATP}$ is the activation constant for ATP.

The flux of oxidative stress due to excess dopamine in the cytoplasm $\left(J_{dopa}\right)$ is given by,

$$J_{dopa} = k_{dopa} * \frac{[DA_c]}{[DA_c] + \left[K_{dopa}\right]} \quad (109)$$

where, k_{dopa} is the reaction constant for ROS production by excess dopamine, $[DA_c]$ is the cytoplasmic dopamine concentration, and K_{dopa} is the affinity constant for $[DA_c]$.

The catabolizing flux of ROS by catalase enzyme (J_{cat}) is given by,

$$J_{cat} = k_{cat} * [ROS] \tag{110}$$

where, k_{cat} is the reaction constant for catalase, and $[ROS]$ is the ROS concentration.

The synthesizing flux of alpha-synuclein protein (J_{syn}) is given by,

$$J_{syn} = k_{syn} \tag{111}$$

where, k_{syn} is the reaction constant for alpha-synuclein synthesis.

The flux of alpha-synuclein misfolding due to ROS (J_{ox}) is given by,

$$J_{ox} = k_{ox} * [ASYN] * [ROS] \tag{112}$$

where, k_{ox} is the reaction constant for alpha-synuclein oxidation, $[ASYN]$ is the alpha-synuclein concentration, and $[ROS]$ is the ROS concentration.

The usage flux of alpha-synuclein in other processes (J_{to}) is given by,

$$J_{to} = k_{to} * [ASYN] \tag{113}$$

where, k_{to} is the reaction constant for alpha-synuclein consumption, and $[ASYN]$ is the alpha-synuclein concentration.

The flux of alpha-synuclein aggregation (J_{agg}) is given by,

$$J_{agg} = k_{agg} * [ASYN_{mis}] * \frac{[ASYN_{mis}]^6}{[ASYN_{mis}]^6 + K_{agg}^6} \tag{114}$$

where, k_{agg} is the reaction constant for alpha-synuclein aggregation, $[ASYN_{mis}]$ is the misfolded alpha-synuclein concentration, and K_{agg} is the affinity constant for $[ASYN_{mis}]$.

The flux of ATP-dependent ubiquitination of damaged protein for proteasomal degradation (J_{tag}) is given by,

$$J_{tag} = k_{tag} * ASYN_{mis} * [Ub] * [ATP_i] \tag{115}$$

$$[Ub] = [Ub_{tot}] - [ASYN_{tag}] \tag{116}$$

where, k_{tag} is the reaction constant for ubiquitination of damaged protein, $[ASYN_{mis}]$ is the misfolded alpha-synuclein concentration, $[Ub]$ is the ubiquitin concentration, $[ATP_i]$ is the intracellular ATP concentration, $[Ub_{tot}]$ is the total ubiquitin concentration, and $[ASYN_{tag}]$ is the tagged alpha-synuclein concentration.

The flux of ATP-dependent breakdown of damaged protein through proteasomal degradation (J_{prt}) is given by,

$$J_{prt} = k_{prt} * [ASYN_{tag}] * [ATP_i] * \left(1 - \beta_{prt} * \left(\frac{[ASYN_{agg}]^4}{[ASYN_{agg}]^4 + K_{prt}^4}\right)\right) \tag{117}$$

where, k_{prt} is the reaction constant for damaged protein disposal by the proteasome, $[ASYN_{tag}]$ is the tagged alpha-synuclein concentration, $[ATP_i]$ is the intracellular ATP concentration, $[ASYN_{agg}]$ is the aggregated alpha-synuclein concentration, K_{prt} is the affinity constant for $[ASYN_{agg}]$, and β_{prt} is the fraction reduction of proteasome activity by $[ASYN_{agg}]$.

The flux of ATP-dependent breakdown of aggregated protein through lysosomal degradation (J_{lyso}) is given by,

$$J_{lyso} = k_{lyso} * [ASYN_{agg}] * [ATP_i] \tag{118}$$

where, k_{lyso} is the reaction constant for $[ASYN_{agg}]$ disposal by the lysosome, and $[ATP_i]$ is the intracellular ATP concentration.

The flux of LB formation (J_{lb}) is given by,

$$V_{lb} = k_{lb} * [ASYN_{agg}] * \frac{[ASYN_{agg}]^6}{[ASYN_{agg}]^6 + K_{lb}^6} \quad (119)$$

where, k_{lb} is the reaction constant for Lewy bodies from $[ASYN_{agg}]$, $[ASYN_{agg}]$ is the aggregated alpha-synuclein concentration, and K_{prt} is the affinity constant for $[ASYN_{agg}]$.

Table S9.1: Parameter values of PD pathology pathways of SNc cell model (Cloutier and Wellstead, 2012).

Constant	Symbol	Value	Units
Activation constant for ATP	$K_{a,leak}$	0.5282	mM
Reaction constant for ROS production due to excess dopamine	k_{dopa}	$4.167\ x\ 10^{-4}$	$mM^{-1} * ms^{-1}$
Affinity constant for $[DA_c]$	K_{dopa}	8.5	mM
Reaction constant for catalase	k_{cat}	$2.35\ x\ 10^{-5}$	ms^{-1}
Reaction constant for alpha-synuclein oxidation	k_{syn}	$1.39\ x\ 10^{-8}$	$mM * ms^{-1}$
Reaction constant for alpha-synuclein consumption	k_{to}	$1.39\ x\ 10^{-7}$	ms^{-1}
Reaction constant for alpha-synuclein aggregation	k_{agg}	$2.08\ x\ 10^{-10}$	ms^{-1}
Affinity constant for $ASYN_{mis}$	K_{agg}	$7.5\ x\ 10^{-3}$	mM
Reaction constant for tagging of damaged protein	k_{tag}	$7.64\ x\ 10^{-11}$	$mM^{-1} * ms^{-1}$
Total ubiquitin concentration	$[Ub_{tot}]$	$10.5\ x\ 10^{-3}$	mM
Reaction constant for damaged protein disposal by the proteasome	k_{prt}	$2.08\ x\ 10^{-10}$	ms^{-1}
Affinity constant for $ASYN_{agg}$	K_{prt}	$5\ x\ 10^{-3}$	mM

Fraction reduction of proteasome activity by $ASYN_{agg}$	β_{prt}	0.25	$dimensionless$
Reaction constant for $ASYN_{agg}$ disposal by lysosome	k_{lyso}	$2.08\ x\ 10^{-11}$	ms^{-1}
Reaction constant for Lewy bodies from $ASYN_{agg}$	k_{lb}	$2.08\ x\ 10^{-11}$	ms^{-1}
Affinity constant for $ASYN_{agg}$	K_{lb}	$5\ x\ 10^{-3}$	mM

Table S9.2: Steady state values of PD pathology pathways of SNc cell model (Cloutier and Wellstead, 2012).

Symbol	Value	Symbol	Value
$[ROS]$	$1\ x\ 10^{-3}\ mM$	$[ASYN_{tag}]$	$1\ x\ 10^{-5}\ mM$
$[ASYN]$	$0.1\ mM$	$[ASYN_{agg}]$	$0\ mM$
$[ASYN_{mis}]$	$1\ x\ 10^{-3}\ mM$	$[LB]$	$0\ mM$

REFERENCES:

Alzheimer, C. (2003). "Na+Channels and Ca2+ Channels of the Cell Membrane as Targets of Neuroprotective Substances," in *Advances in Experimental Medicine and Biology*, 161–181. doi:10.1007/978-1-4615-0123-7_5.

Anzalone, A., Lizardi-Ortiz, J. E., Ramos, M., De Mei, C., Hopf, F. W., Iaccarino, C., et al. (2012). Dual control of dopamine synthesis and release by presynaptic and postsynaptic dopamine D2 receptors. *J. Neurosci.* 32, 9023–9024. doi:10.1523/JNEUROSCI.0918-12.2012.

Buxton, D., Bracci, E., Overton, P. G., and Gurney, K. (2017). Striatal Neuropeptides Enhance Selection and Rejection of Sequential Actions. *Front. Comput. Neurosci.* 11, 62. doi:10.3389/fncom.2017.00062.

Chen, R., Wei, J., Fowler, S. C., and Wu, J.-Y. (2003). Demonstration of functional coupling between dopamine synthesis and its packaging into synaptic vesicles. *J. Biomed. Sci.* 10, 774–781. doi:10.1007/BF02256330.

Cloutier, M., and Wellstead, P. (2012). Dynamic modelling of protein and oxidative metabolisms simulates the pathogenesis of Parkinson's disease. *IET Syst. Biol.* 6, 65–72. doi:10.1049/iet-syb.2011.0075.

Destexhe, A., Mainen, Z. F., and Sejnowski, T. J. (1998). Kinetic models of synaptic transmission. *Methods Neuronal Model.*, 1–25. Available at: http://cns.iaf.cnrs-gif.fr/abstracts/KSchap96.html.

Ford, C. P. (2014). The role of D2-autoreceptors in regulating dopamine neuron activity and transmission. *Neuroscience* 282, 13–22. doi:10.1016/j.neuroscience.2014.01.025.

Francis, F., García, M. R., and Middleton, R. H. (2013). A single compartment model of pacemaking in dissasociated substantia nigra neurons: stability and energy analysis. *J. Comput. Neurosci.* 35, 295–316. doi:10.1007/s10827-013-0453-9.

Humphries, M. D., Lepora, N., Wood, R., and Gurney, K. (2009). Capturing dopaminergic modulation and bimodal membrane behaviour of striatal medium spiny neurons in accurate, reduced models. *Front. Comput. Neurosci.* 3, 26. doi:10.3389/neuro.10.026.2009.

Izhikevich, E. M. (2003). Simple model of spiking neurons. *IEEE Trans. Neural Networks* 14, 1569–1572. doi:10.1109/TNN.2003.820440.

Marhl, M., Haberichter, T., Brumen, M., and Heinrich, R. (2000). Complex calcium oscillations and the role of mitochondria and cytosolic proteins. *BioSystems* 57, 75–86. doi:10.1016/S0303-2647(00)00090-3.

Muddapu, V. R., and Chakravarthy, V. S. (2021). Influence of energy deficiency on the subcellular processes of Substantia Nigra Pars Compacta cell for understanding Parkinsonian neurodegeneration. *Sci. Rep.* 11, 1754. doi:10.1038/s41598-021-81185-9.

Muddapu, V. R., Mandali, A., Chakravarthy, V. S., and Ramaswamy, S. (2019). A Computational Model of Loss of Dopaminergic Cells in Parkinson's Disease Due to Glutamate-Induced Excitotoxicity. *Front. Neural Circuits* 13, 11. doi:10.3389/fncir.2019.00011.

Oorschot, D. E. (1996). Total number of neurons in the neostriatal, pallidal, subthalamic, and substantia nigral nuclei of the rat basal ganglia: A stereological study using the cavalieri and optical disector methods. *J. Comp. Neurol.* 366, 580–599. doi:10.1002/(SICI)1096-9861(19960318)366:4<580::AID-CNE3>3.0.CO;2-0.

Reed, M. C., Nijhout, H. F., and Best, J. A. (2012). Mathematical Insights into the Effects of Levodopa. *Front. Integr. Neurosci.* 6, 1–24. doi:10.3389/fnint.2012.00021.

Tello-Bravo, D. (2012). A Mathematical Model of Dopamine Neurotransmission. *ASU Libr.* Thesis. Available at: https://repository.asu.edu/items/14832.

Terman, D., Rubin, J. E., Yew, a C., and Wilson, C. J. (2002). Activity patterns in a model for the subthalamopallidal network of the basal ganglia. *J. Neurosci.* 22, 2963–76. doi:20026266.

Venda, L. L., Cragg, S. J., Buchman, V. L., and Wade-Martins, R. (2010). α-Synuclein and dopamine at the crossroads of Parkinson's disease. *Trends Neurosci.* 33, 559–568. doi:10.1016/j.tins.2010.09.004.
